# Shallow-water mussels (*Mytilus galloprovincialis*) adapt to deep-sea environment through transcriptomic and metagenomic insights

**DOI:** 10.1038/s42003-024-07382-0

**Published:** 2025-01-14

**Authors:** Luyang Sun, Xiaolu Liu, Li Zhou, Hao Wang, Chao Lian, Zhaoshan Zhong, Minxiao Wang, Hao Chen, Chaolun Li

**Affiliations:** 1 Single-Cell Center, CAS Key Laboratory of Biofuels, Shandong Key Laboratory of Energy Genetics, Shandong Energy Institute, Qingdao New Energy Shandong Laboratory, Qingdao Institute of Bioenergy and Bioprocess Technology, Chinese Academy of Sciences, 266101 Qingdao, China; 2 Laboratory for Marine Biology and Biotechnology, Qingdao Marine Science and Technology Center, 266104 Qingdao, China; 3 University of Chinese Academy of Sciences, 10049 Beijing, China; 4 Center of Deep Sea Research, Institute of Oceanology, Chinese Academy of Sciences, 266071 Qingdao, China; 5 CAS Key Laboratory of Marine Ecology and Environmental Sciences, Institute of Oceanology, Chinese Academy of Sciences, 266071 Qingdao, China; 6 South China Sea Institute of Oceanology, Chinese Academy of Sciences, 510301 Guangzhou, China

**Keywords:** Experimental evolution, Evolutionary ecology, Symbiosis, Evolutionary biology, Data integration

## Abstract

Recent studies have unveiled the deep sea as a rich biosphere, populated by species descended from shallow-water ancestors post-mass extinctions. Research on genomic evolution and microbial symbiosis has shed light on how these species thrive in extreme deep-sea conditions. However, early adaptation stages, particularly the roles of conserved genes and symbiotic microbes, remain inadequately understood. This study examined transcriptomic and microbiome changes in shallow-water mussels *Mytilus galloprovincialis* exposed to deep-sea conditions at the Site-F cold seep in the South China Sea. Results reveal complex gene expression adjustments in stress response, immune defense, homeostasis, and energy metabolism pathways during adaptation. After 10 days of deep-sea exposure, shallow-water mussels and their microbial communities closely resembled those of native deep-sea mussels, demonstrating host and microbiome convergence in response to adaptive shifts. Notably, methanotrophic bacteria, key symbionts in native deep-sea mussels, emerged as a dominant group in the exposed mussels. Host genes involved in immune recognition and endocytosis correlated significantly with the abundance of these bacteria. Overall, our analyses provide insights into adaptive transcriptional regulation and microbiome dynamics of mussels in deep-sea environments, highlighting the roles of conserved genes and microbial community shifts in adapting to extreme environments.

## Abstract (web summary)

In situ exposure of shallow-water mussels to deep-sea conditions induced changes in their transcriptome and microbiome, with profiles gradually aligning with those of native deep-sea mussels, highlighting the conserved interplay between evolutionary adaptations and rapid adjustments.

## Introduction

The deep sea, which lies at depths of over 200 m, is the Earth’s most extensive environment, encompassing over 90% of the total surface area of the world’s oceans. Previously considered barren and lifeless due to its darkness, low temperatures, and high pressures, it is now widely recognized as a rich hub of biodiversity, home to an incredible array of ecosystems and species^1^. The deep-sea faunas are regarded to have evolved from ancestors that once inhabited shallow waters as a result of multiple mass extinction events throughout the Phanerozoic era^2^. Accordingly, organisms from shallow waters had to endure various stress factors before being able to settle and thrive in the deep-sea environment. Understanding the genetic basis of the physiological adaptations that allowed shallow-water organisms to repeatedly infiltrate and adapt to different deep-sea environments, ultimately resulting in the current astonishing biodiversity, is an important scientific pursuit for deep-sea biologists and ecologists.

Mytilidae mussels are widely distributed and have adapted to various habitats ranging from shallow water to the deep-sea^3^. Shallow-water mytilids are often used as environmental sentinels due to their extreme tolerance to various environmental stresses^4^. For instance, extensive research has been conducted on the response mechanisms of various abiotic stresses in the environment^5–7^. Deep-sea mytilids of the subfamily Bathymodiolinae, meanwhile, are one of the dominant species found at hydrothermal vents and cold seeps. In addition to their unique abilities to withstand various abiotic stresses like high pressure, low temperature and darkness, they can also build symbiotic relationships with deep-sea bacteria to obtain food and energy^8^. Their wide distribution and ability to withstand different environmental conditions underscore their strong genetic potential for adaptation. As such, mussels serve as an ideal model species for investigating the mechanisms underlying their successful acclimatization to deep-sea environments.

Numerous studies have revealed that shallow-water mytilids gradually adapted to cold seeps over millions of years, eventually adapting to deep-sea vents. Craddock et al. ^9^ were among the first to support the theory of mussel transition from shallow water to deep-sea habitats using allozyme and morphology-based phylogenetic analysis^9^. Subsequently, several studies showed that mussels progressively adapted to deep-sea environments by exploiting resources like sunken wood and whale carcasses, through comparing mitochondrial genes between *Bathymodiolus* mussels and their mytilid relatives^10–12^. The diversification of bathymodioline mussels began in the early Miocene, with the groups undergoing further diversification in the early to middle Miocene^11^. Most recently, Sun et al. ^13^ conducted comparative genomics between the deep-sea mussel *Bathymodiolus platifrons* (current name: *Gigantidas platifrons*; NCBI Taxonomy ID: 2830794) and its shallow-water counterpart *Modiolus philippinarum*, revealing that the deep-sea mussel evolved from its shallow-water ancestor while retaining most genomic content^13^. These studies provide strong genomic evidence supporting the evolutionary process of mussel adaptation from shallow water to deep-sea environments mainly through phylogenetic analyses. However, direct evidence of the adaptation process to the deep-sea environment, especially from in situ studies, remains limited.

Among the many stress factors of deep-sea environment, hydrostatic pressure is one of the primary environmental factors which limits the vertical distribution of marine species from shallow to the deep and is the most extensively studied. Many studies have been conducted to simulate the deep-sea hydrostatic pressure in laboratory, aiming to investigate the genetic basis of deep-sea adaption. These studies have revealed that genes involved in stress response, antioxidation, energy metabolism and DNA repair are induced under lab-simulated high hydrostatic pressure and low temperatures among the various species, including *Palaemonetes varians* (shrimp)^14^, *Eogammarus possjeticus* (amphipod)^15^, *Apostichopus japonicus* (sea cucumber)^16,17^, and *Danio rerio* (zebrafish)^18^. However, the deep-sea ecosystem is an integrated system that involves not only hydrostatic pressure but also a complete set of abiotic and biotic factors, such as local microbial communities, cold seep activities, sulfide concentrations, and other physicochemical conditions. Consequently, deep-sea species often leverage symbiotic relationships with microorganisms to better adapt and thrive in the challenging conditions of the deep sea. For example, Bathymodiolinae mussels take advantage of chemoautotrophic endosymbionts that colonize their specialized gill epithelium (bacteriocytes) to oxidize reduced chemical compounds and obtain nutrition from the environment^19–21^. Lastly, even in situ deep-sea experiments, if conducted without in situ RNA fixation, could introduce bias, as evidenced by the case of comparison between the onboard fixation and in situ fixation. They reported that the concomitant stress during conventional deep-sea sampling without RNA in situ fixation can greatly influence the gene expression related to carious biological processes, including cell and tissue structure, lysosomal activity, fluid balance, and unsaturated fatty acid metabolism^22^. Thus, in situ deep-sea experiments and fixation are necessary to better capture the organisms’ most primitive physiological states and the underlying transcriptomic changes during the deep-sea adaptation process.

In this study, we investigated the mechanisms underlying deep-sea adaptation in Mytilidae mussels by transplanting shallow-water *Mytilus galloprovincialis* to a depth of 1119 m in the South China Sea. The mussels were exposed to the deep-sea environment for 6 h and 10 days, serving as the short and long-term simulation of the adaption process of shallow-water mussels into deep-sea environments. Both transcriptional and microbial composition changes in the gill tissues associated with deep-sea adaptation were examined. Our results demonstrated that the deep-sea environment induced time-dependent transcriptional changes in the shallow-water mussels, progressively aligning their transcription profiles with those of native deep-sea mussel species. Notably, we observed a significant presence of methanotrophic bacteria in the long-term deep-sea-exposed mussels, resulting in a microbial community similar to that of native deep-sea mussels. These findings suggest that, in contrast to the evolutionary timescale of tens of millions of years required for shallow-water mytilids to evolve into deep-sea mytilids, shallow-water mussels can adjust their transcriptome to resemble that of native deep-sea mussels within just 10 days. This rapid adaptation and the associated microbial community changes underscore the plasticity and resilience of mussels in response to extreme environmental shifts, providing insights into the initiation and development of symbiotic relationships between deep-sea mussels and their functional microbiomes.

## Results

### Deep-sea environment profoundly shaped the transcriptome profile of shallow-water mussels

**Fig. 1:**
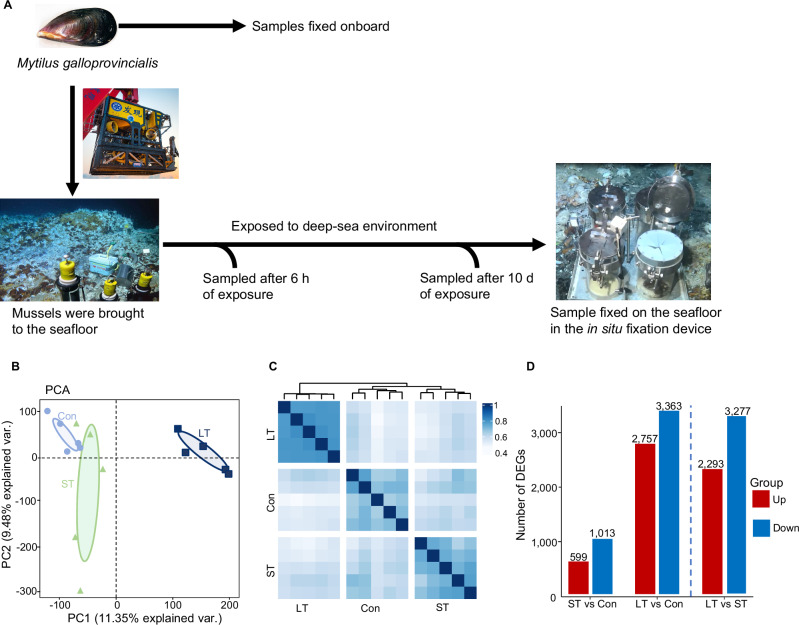
Experiment designs and comprehensive analysis of gene expression patterns. **A** Experiment designs. Samples of the control group (Con) were cultured onboard, samples in short-term pressure (ST) and long-term pressure (LT) groups were exposed to the seabed at Site-F cold seep for 6 h and 10 days, respectively. Following exposure, samples exposed to the deep-sea were in situ sampled and fixed on-site. **B** Principal Component Analysis (PCA) of all gene expressions (Confidence ellipses level = 0.9). **C** Samples correlation heat map with hierarchical clustering from the differentially expressed genes (DEGs). **D** Histogram showing the number of DEGs in the three comparisons (red: up-regulated genes; blue: down-regulated genes).

To explore adaptation mechanisms to the deep sea, shallow-water mussels *M. galloprovincialis* adapted to the onboard environment were deployed on the seabed of Site-F cold seep in the South China sea (Supplementary Fig. 1; 22°06.919’N and 119°17.140’E; 1119 m deep) for 6 h (short time period, ST) and 10 days (long time period, LT), and fixed on-site. Mussels onboard without deep-sea exposure were fixed onboard and used as controls (Con, Fig. 1A). Mortality assessments of the deep-sea-exposed mussels, using the non-fixed ones, indicated a 100% survival rate. This was evidenced by their tightly closed shells and subsequent dissections confirming all tissues were intact and undisturbed, affirming their robust survival state. The initial RNA-seq results showed that the de novo transcriptome assembly produced 495,264 unigenes with an Ex90N50 length of 1780 bp, with a median read mapping of approximately 86.41% (Supplementary Table 1; Supplementary Table 2). The quality of the final assembly was further assessed through BUSCO against the Eukaryota database, resulting in a 99.2% BUSCO completeness value (Supplementary Fig. 2), then the assembly was functionally annotated followed the Trinotate protocol (See Methods).

To comprehensively understand gene expression patterns over time, we performed Principal Component Analysis (PCA) first. The PCA results showed that the control and short-term groups clustered closely, whereas the long-term group formed a distinct cluster, demonstrating that the deep-sea environment markedly altered the transcriptomic profile of shallow-water mussels, particularly over an extended period (Fig. 1B). Correlation analysis supported these findings, showing close relationships between control and short-term samples, while the long-term samples branched off into a separated clade (Fig. 1C). These results suggest that the gene expression profiles of the short-term group remained relatively similar to those of the control, yet underwent considerable regulation. Notably, the short-term (ST) group exhibited noticeable dispersion along the second principal component (PC2, Fig. 1B). Hierarchical clustering analysis confirmed high reproducibility within each condition (Fig. 1C), suggesting that the variability likely reflects biological adaptation rather than technical artifacts.

To further investigate gene expression dynamics, we analyzed differentially expressed genes (DEGs) across the three groups. Our findings indicate a predominance of down-regulated genes in response to the deep-sea environment, with an increasing number of DEGs identified over time (Fig. 1D). Notably, a comparison between the short- and long-term groups revealed significant transcriptomic alterations over the duration of exposure, suggesting a progressive and time-dependent genetic response to the deep-sea environment. In summary, our findings indicate that shallow-water mussels exhibit temporal alterations in gene expression when exposed to deep-sea conditions. Unlike the control and long-term exposure groups, which maintained stable expression patterns, mussels subjected to short-term deep-sea conditions demonstrated varied expression profiles, indicating personalized adaptive responses to deep-sea stressors.

### Rapid immune and homeostatic responses combined with gradual metabolic adjustments facilitate deep-sea environmental adaptation in mussels

**Fig. 2:**
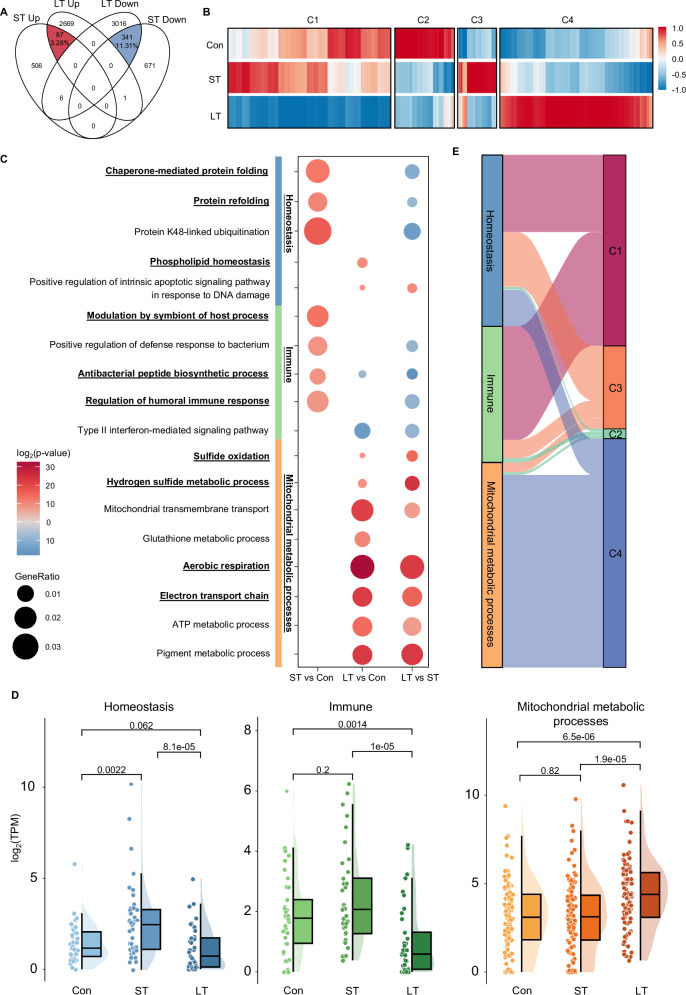
Dynamics of transcriptomic and functional enrichment analysis. **A** Venn diagram shows the number of shared and unique DEGs among the different exposure durations. **B** The heat map depicting all DEGs divided into four clusters (k-means = 4). **C** Gene Ontology (GO) biological process enrichment analysis depicting the interactions among all comparisons (red: enriched using up-regulated DEGs; blue: enriched using down-regulated DEGs). Based on the characteristics of significantly enriched GO terms, we categorized terms into three groups. **D** Expression boxplot of DEGs involved in significantly enriched GO categories, with group comparisons conducted using the Wilcoxon rank sum test. **E** Alluvial plot exhibiting the relationships between GO categories and the expression clusters.

To comprehensively understand the functional dynamics of adaptation to the deep-sea environment over time, we examined the changes in expression patterns and enriched pathways across the groups. The Venn diagram revealed limited overlap in DEGs between short- and long-term groups, especially among the up-regulated genes (Fig. 2A), suggesting distinct genetic responses to the deep-sea environment over time. All identified DEGs were analyzed and categorized into four clusters based on their expression patterns (Fig. 2B). The majority of DEGs clustered into C1 and C4, showing divergent expression trends between the long-term and other groups. C1 genes were down-regulated only in the long-term group, while C4 genes were up-regulated exclusively. In contrast, C3 genes activated only in the short-term group, and C2 genes were consistently repressed in the deep-sea environment. These expression patterns highlight the varied roles of gene functional modules and the prioritization of distinct responses throughout deep-sea adaptation phases.

We next analyze and contrast the enriched biological processes in the three comparisons (ST vs Con, LT vs Con, and LT vs ST), revealed that the Gene Ontology (GO) terms associated with homeostasis, immunity, and mitochondrial metabolic processes were significantly enriched (Fig. 2C, D). In the short-term deep-sea exposure, mussels rapidly activated protein quality control mechanisms, as evidenced by the upregulation of genes related to chaperone-mediated protein folding and refolding, to maintain homeostasis in the deep-sea environment (Fig. 2C). Specifically, genes associated with protein maintenance chaperones (HSP and DNAJ) showed significant activation within 6 h before returning to baseline levels (Supplementary Fig. 3A). Over long-term exposure, pathways related to phospholipid homeostasis and intrinsic apoptotic signaling in response to DNA damage were enriched. Interestingly, the apoptosis inhibitor genes IAP and BIR were significantly activated in the short-term response to the deep-sea environment, prior to the activation of the DNA damage-induced apoptotic pathway (Supplementary Fig. 3A). These results suggest a potential survival strategy for shallow-water mussels in deep-water environment that initially involves activating apoptosis inhibitor genes IAP and BIR to mitigate immediate high-pressure effects, allowing for DNA repair attempts. If damage remains, the cell shifts towards apoptosis to eliminate damaged cells, balancing survival with genomic integrity under stress. Additional analysis showed that genes involved in these homeostasis pathways were predominantly belonged to gene expression pattern C1 and C3 (Fig. 2B, E), confirming that gene expression was mainly upregulated in the short term and downregulated in the long term.

In addition to the dynamic pattern of homeostasis-associated genes, immune-related GO terms such as regulation of humoral immune response, modulation by symbiont of host process, and antibacterial peptide biosynthetic process were specifically enriched in the short-term group (Fig. 2C, D). High concentrations of methane and sulfides at Site-F cold seep support a unique microbial community dominated by anaerobic methanotrophic archaea and sulfate-reducing bacteria^23–26^. This sudden exposure to the deep-sea microbial community (biological stressors), along with environmental stressors (non-biological) like high hydrostatic pressure, likely triggered immune and stress responses in mussels during their short-term adaptation. Notably, the activation of stress and immune responses was transient. When mussels continued to stay in the deep sea for 10 days, the activation of stress and immune responses stabled, indicating the mussels’ adaptation to the deep-sea environment (Fig. 2C, D). Most immunity-related genes belonged to gene expression pattern C1 and C3 (Fig. 2E), showing an initial upregulation short-term, followed by downregulation in long-term. In summary, the similar dynamic patterns of genes between homeostasis and immunity suggest that mussels activated a series of mechanisms in response to non-biological and biological stresses when sudden exposure to the deep-sea environment.

In the long-term exposure to the deep-sea environment, instead of the activation of homeostasis and stress-related genes, genes involved in multiple mitochondrial metabolic processes were activated. These include the oxidative and metabolic processing of sulfides, mitochondrial respiration, and the electron transport chain (Fig. 2C, D, and (Supplementary Fig. 4). Furthermore, pathways centered on the TCA cycle, such as the metabolic processes of tricarboxylic acid, pyruvate, and citrate, were also activated (Supplementary Fig. 3B). The activation of these metabolic pathways suggests that the mussels were undergoing metabolic adjustments to better utilize available resources and manage potential toxins, such as sulfides, in the deep-sea environment. Moreover, most genes involved in mitochondrial metabolic processes were clustered in C4, showing unique activation in the long-term treatment group (Fig. 2B, E). Overall, the enhancement in energy production and utilization indicates an adaptation strategy to meet the increased energy demands characteristic of the challenging deep-sea conditions.

### The gene expression profile of deep-sea adapted shallow-water mussels substantially resembles that of native deep-sea species

**Fig. 3:**
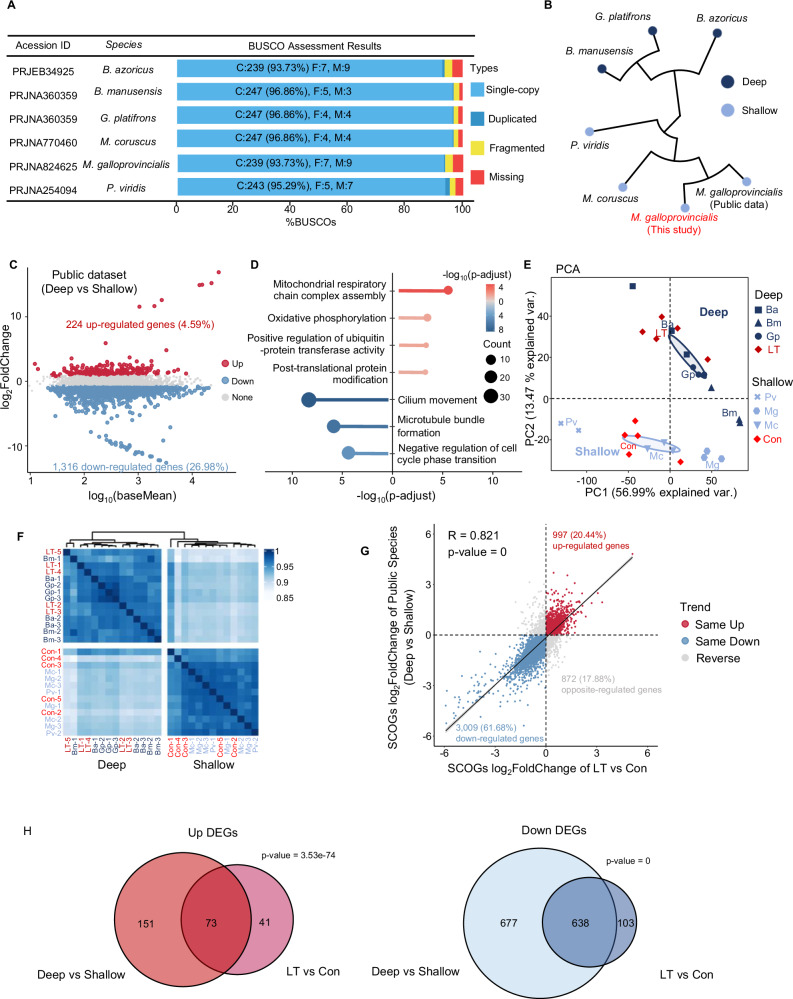
Phylotranscriptomic analysis using seven species from experimental and public mussels. **A** Metadata for public data used in this study, including the NCBI BioProject accession numbers, taxonomic names, and the BUSCO completeness assessments of the de novo assemblies. **B** Phylogenetic tree of seven species inferred from the single-copy orthologous genes (SCOGs) identified by sequences of open reading frames (ORFs) among all reference transcriptomes. **C** MA plot showing differentially expressed SCOGs (DESCOGs) between deep- and shallow-species from public datasets (red: up-regulated DESCOGs; blue: down-regulated DESCOGs; gray: non-significantly DESCOGs). **D** GO biological process enrichment analysis using identified DESCOGs; enriched GO terms with p-adjust < 0.05 were considered significantly enriched. PCA (**E**) and correlation heat map (**F**) of all samples from experimental and public mussels with batch effect correction, except for ST groups (Confidence ellipses level = 0.9). Ba: *Bathymodiolus azoricus*; Bm: *B. manusensis*; Gp: *Gigantidas platifrons* (basionym: *B. platifrons*); Mc: *Mytilus coruscus*; Mg: *M. galloprovincialis*; Pv: *Perna viridis*. **G** Scatter plot showing the log_2_FoldChange Pearson Correlation Coefficient (*R*) of all SCOGs between LT vs. Con and Deep vs. Shallow. One SCOG was removed due to NA generated from fold change calculation. **H** Venn diagrams showing the numbers of shared DESCOGs in experimental and public comparisons (left: up-regulated DESCOGs; right: down-regulated DESCOGs). Co-occurrence of the intersections was tested using two-sided Fisher’s exact test.

Previous findings suggest that to adapt to the deep-sea environment, shallow-water mussels gradually alter their gene expression profiles in a time-dependent manner. However, it is unclear whether these changes indicate a shift towards the expression patterns characteristic of native deep-sea species. To assess the similarity in transcriptomic profiles between long-term adapted shallow-water mussels and native deep-sea mussels, we conducted a comprehensive comparative phylotranscriptomic analysis across a range of shallow-water and deep-sea mussel species. A total of 17 publicly available datasets were obtained from six species (Supplementary Table 3), including three deep-sea species (*B. azoricus*, *B. manusensis*, *G. platifrons*) and three shallow-water species (*M. coruscus*, *M. galloprovincialis*, *Perna viridis*). We assembled reference transcriptomes for each species using parameters consistent with our own data and evaluated them through BUSCO, achieving a minimum BUSCO completeness value of 93% (Fig. 3A).

Before determining the phylogenetic relationships among gene sequences, single-copy orthologous genes (SCOGs) were identified, which provides a framework for understanding evolution and facilitates the extrapolation of biological knowledge between organisms^27^. Orthogroups were computed for all seven reference transcriptomes, with 84.4% of genes being successfully assigned to these groups, and 4878 single-copy orthologous genes (SCOGs) were identified. The phylogeny of these mussels was reconstructed from an alignment of all SCOGs, supporting the adaptive radiation phylogenetic relationship (Fig. 3B). As expected, shallow- and deep-species were clustered into separate branches, with our studied mussels *M. galloprovincialis* located within the shallow-water species, close to public dataset of the species *M. galloprovincialis*, confirmed the accuracy of the phylogeny. Notably, the phylogeny inferred from transcriptome based SCOGs was largely in agreement with those inferred from comparative genomic studies^13^.

Previous phylogenomic analyses indicated that the deep-sea mussel *G. platifrons* retained most of the genes from its shallow-water ancestors^13^. Given the evolutionary and functional conservation of SCOGs, we then examined the differences in baseline expressions between native shallow- and deep-sea species. The results revealed that a total of 1540 (31.57%) SCOGs were differentially expressed, including 224 (4.59%) significantly up-regulated and 1316 (26.98%) down-regulated genes (Fig. 3C). Subsequent GO enrichment analysis of these differentially expressed SCOGs (DESCOGs) were then performed among the native-species (Fig. 3D). The up-regulated SCOGs were significantly enriched in functions related to mitochondrial metabolisms, protein degradation, and modification, aligning with our previous findings (Fig. 2C) that genes involved in mitochondrial metabolic processes were up-regulated in mussels exposed to the deep-sea environment over the long term. Conversely, the enrichment of down-regulated SCOGs pointed to a decrease in genes associated with cilium movement, cytoskeleton and cell cycle, suggesting a significant repression of microtubule cytoskeleton organization and cilium movement for mussels in the deep-sea environment (Fig. 3D). Notably, these genes are specifically expressed in shallow-water mussels, in contrast to their deep-sea counterparts^13^. To obtain clear results in exploring the dynamic patterns between our long-term experiments and native species, we performed PCA analysis on data from the control group (Con), the long-term deep-sea treatment group (LT), and with public datasets. Batch effect corrections were conducted before the analysis. The PCA results demonstrated distinct evolutionary divergences in baseline gene expressions between shallow- and deep-sea species, leading to their significant bifurcation into two clusters (Fig. 3E). Notably, mussels from the control group aligned with shallow-water species, while those from the long-term group closely clustered with native deep-sea species. This demonstrates the similarity in global gene expression between shallow-water mussels exposed to long-term deep-sea conditions and native deep-sea mussels. Interestingly, when including short-term deep-sea treatment group (ST) in the PCA analysis (Supplementary Fig. 5), it not only exhibited the expression similarity between the long-term and native deep-sea mussels but also preserved the differences among the experimental groups that dispersed along PC2, consisted with the trend depicted in Fig. 1B. This result further demonstrated that, to adapt to the deep-sea environment, mussels gradually regulated their gene expression profiles to mimic that of native deep-sea mussels. In line with the PCA results, gene expression correlation analysis showed that mussels in the long-term deep-sea environment clustered with deep-species in the same cluster branch (Fig. 3F). Furthermore, global comparisons of expression patterns between long-term adapted and native species revealed a clear positive correlation in expression changes of SCOGs during long-term deep-sea adaptation and their gene expression changes in native species (Pearson Correlation Coefficient = 0.821; Fig. 3G). When only examining the shared DESCOGs between the experimental and native comparisons, an even higher positive correlation was observed (Pearson Correlation Coefficient = 0.884; Supplementary Fig. 6). Specifically, 64.0% (73) up-regulated DESCOGs in long-term were also up-regulated in native deep-sea species, and 86.1% (638) down-regulated DESCOGs in long-term were also down-regulated (Fig. 3H). Overall, these data compellingly showed that long-term deep-sea adaptation induces gene expression changes in shallow-water mussels, leading to convergence with the expression patterns of native deep-sea mussels.

### Comprehensive network analysis reveals conserved gene co-expression module underlying deep-sea adaptation

**Fig. 4:**
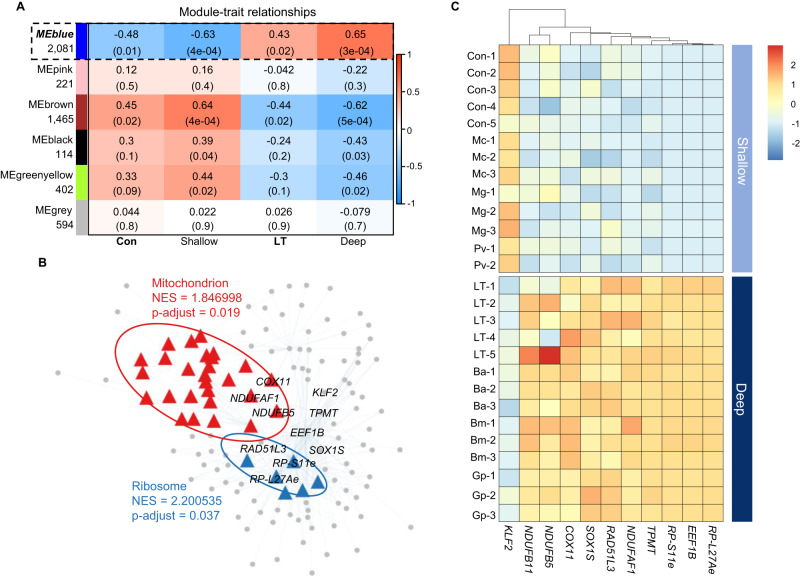
Co-expression analysis using all SCOGs revealed the gene expression convergence with native deep-sea species in long-term exposure. **A** Heat map showing the relationships between modules and sample traits. The numbers of member in each module are presented below the module names. Each cell shows the Pearson correlation coefficient between the module eigengene (row) and the specific sample trait (group in column). Statistically significant relationships were labeled under the correlation coefficient, module with a *p*-value < 0.05 and positive correlation with deep-sea environmental conditions was considered as the candidate module for the further analysis. The candidate module was outlined with dotted line. **B** The visualized co-expression network from the identified candidate module, MEblue. GO Gene Set Enrichment Analysis (GSEA) result presents on the network with the Normalized Enrichment Score (NES) and p-adjust. GO terms with p-adjust < 0.05 were considered as significantly enriched. The hub genes were further identified, labeled in the center of network. *NDUFAF5*, NADH dehydrogenase [ubiquinone] 1 alpha subcomplex assembly factor 5; *ETHE1*, Ethylmalonic Encephalopathy 1; *MRPS34*, Mitochondrial Ribosomal Protein S34; *RP-L27Ae*, Large Subunit Ribosomal Protein L27Ae. **C** Expression heat map of hub genes identified in MEblue module.

The analyses above revealed a robust correlation in gene expression between the mussels from the long-term group and native deep-sea species. Both differential expression analyses comparing long-term vs. control conditions and native deep-sea vs. shallow-water mussels emphasized the critical role of mitochondrial metabolic processes in mussel adaptation to deep-sea environments (Fig. 2C; Fig. 3H). Previous analyses primarily focused on genes with significant expression changes, excluding most genes and potentially overlooking crucial patterns in gene relationships, such as co-expression. To enhance our understanding of the biological mechanisms underlying deep-sea adaptation, we leveraged both our data and publicly available datasets to perform a Weighted Gene Co-expression Network Analysis (WGCNA; refer to Supplementary Fig. 7). We aimed to identify co-expression gene module linked to deep-sea adaptation by classifying the samples into four categories: Control (Con), Shallow, Long-term deep-sea treatment group (LT), and Deep. A total of six color-coded candidate gene modules with dense interconnection^28^ were identified based on their gene expression patterns, with the 42.75% of SCOGs (2081) being assigned to MEblue module (Fig. 4A), which also showed the most significant correlation to environmental conditions, for further investigation (See Methods). The MEblue module positively associated with the deep-sea environment in both experimental and native groups, indicating that it contains genes which are highly expressed in long-term deep-sea treated and native deep-sea mussels, but exhibit low expression in native shallow-water mussels. We then performed Gene Set Enrichment Analysis (GSEA) and identified module hub genes to explore the potential functions embedded in the candidate module. Remarkably, mitochondrion-related genes were significantly enriched in the module that positively correlate with deep-sea conditions (Fig. 4B), which aligns with our previous GO term analyses that mitochondrial metabolic processes contribute to the adaptation of deep sea in long-term by both our data and the published datasets (Figs. 2C,  3H). In addition, genes encoding ribosomal proteins were enriched, indicating that there is a heightened demand for protein synthesis. Previous study of hadal sea cucumber *Paelopatides* sp. Yap showed that high hydrostatic pressure strongly inhibited the protein biosynthesis, resulting the positive selection and expansion of genes associated with ribosomal and translational proteins^29^.

We further identified hub genes within the candidate module, which plays a central role in the co-expression network and often indicating key regulatory functions or biological significance (Fig. 4C)^28^. Our findings highlight genes associated with mitochondria (*NDUFAF1*, *NDUFB10*, *NDUFB9*, *COX11*), ribosome (*RP-L27Ae*, *RP-S11e*, *EEF1B*), and DNA repair (*RAD51L3*) as crucial for mussel adaptation to the deep-sea environment. Additionally, TPMT, known for its role in detoxification^30^, was also identified as one of the hub genes, suggesting its vital role in detoxification of sulfides, contributing to the overall stress response and energy metabolism.

Lastly, we investigated the relationship between adaptive transcriptional regulation and evolution by identifying evolutionarily positively selected genes (PSGs)^31^ among the key regulatory genes explored by GSEA (Fig. 4B, C). A total of 4 PSGs were activate in deep-sea conditions in both our experimental and public datasets, including *NDUFAF5*, *ETHE1*, *MRPS34* and *RP-L27Ae* (Supplementary Data 1). Further examination revealed that these PSGs were functionally involved in mitochondrial respiration^32^, sulfides metabolism^33^ and protein biosynthesis^34,35^. Combining these findings with previous observations of gene expression convergence between experimental and native deep-sea mussels (Fig. 3), these results suggest the possibility that long-term adaptive transcriptional changes may influence the evolutionary trajectory of deep-sea mussels, solidifying their capacity for mitochondrial metabolism, detoxification, and protein synthesis into stable evolutionary adaptations.

### Methanotrophic bacteria had enriched in gills of the deep-sea adapted mussels

Previous studies have shown that intracellular and extracellular chemoautotrophic symbionts play a vital role in deep-sea mussels surviving in diverse hostile environments^8^. These well-established symbiotic relationships provide hosts with nutrition by utilizing energy sources that are typically inaccessible to macrofauna^36^. Deep-sea mussels exemplify this symbiotic relationship, with a mechanism of symbiosis that remains under investigation. It is suggested that the bacterial symbionts of deep-sea mussels may have been acquired from the local environment^37,38^. We previously found that the transcriptomic profile of shallow-sea mussels adapted to long-term deep-sea environments is similar to that of native deep-sea mussels (Fig. 3 and Fig. 4). However, it remained unknown whether these deep-sea adapted mussels could acquire local environmental microbes like the native deep-sea mussels. To explore the dynamics of the microbial community in the gills of shallow-water mussels, we conducted microbial metagenomic sequencing on the gills of both control mussels and long-term adapted mussels using the same samples. Additionally, we obtained publicly available metagenomic data from the gills of cold-seep mussels (*G. platifrons*)^39^, seawater^40^, and seabed sediment^40–42^, which were also sampled from the Site-F cold seep area (Supplementary Table 4).

**Fig. 5:**
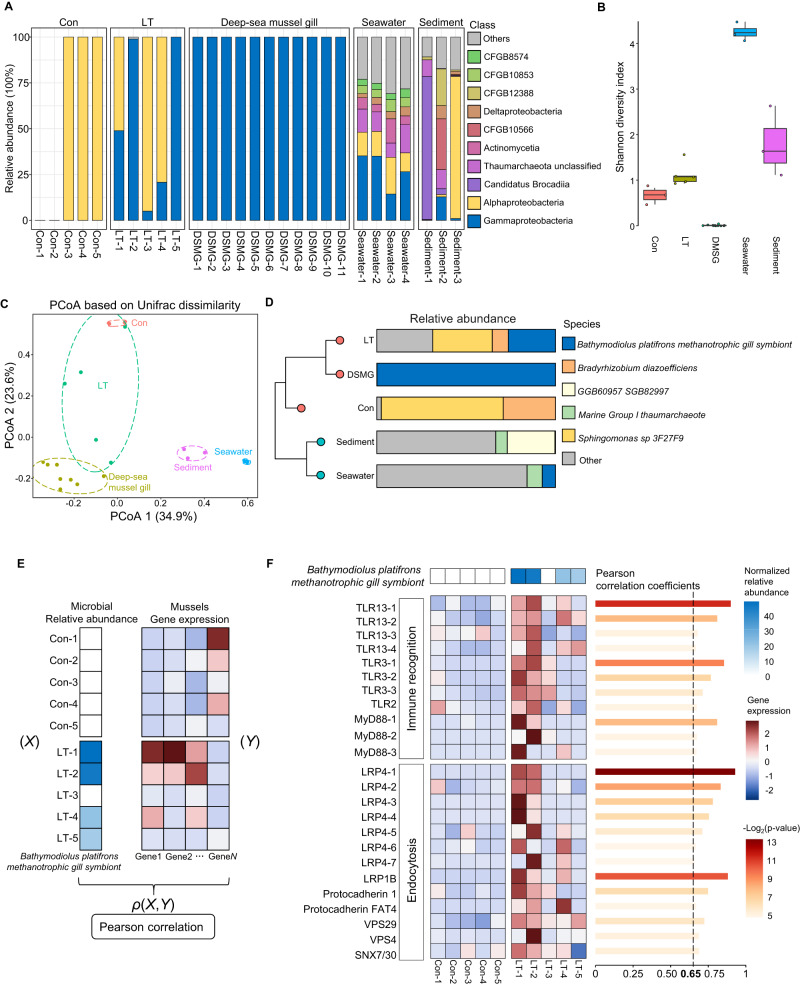
Metagenomic analysis revealed the native symbiont of methanotrophic bacteria enriched in the gills of long-term adapted mussels. **A** Taxonomic composition in relative abundances of the top 10 classes among the experimental and public data. DMSG, deep-sea mussel gill. **B** Shanno diversity indexes among the groups showed differences in species richness and the relative abundances. **C** Unifrac dissimilarity based PCoA exhibited significant differences between the biological and environmental samples, and the high heterogeneity of microbial profiles were observed within LT group. **D** Stacked plot of average abundances showed the differences microbial communities among the groups. The left hierarchical clustering tree was based on Unifac dissimilarity. **E** Schematic diagram of method for filtering the potentially highly correlated genes with presence of methanotrophic bacteria. Computing the Pearson correlation coefficient between the normalized relative abundance of methanotrophic bacteria and each gene expression using all Con and LT samples. **F** The heat map shows the expression levels of highly correlated genes with the abundance of methanotrophic bacteria, with normalized relative abundance indicated above heat map to illustrate their dynamic consistency. Each row corresponds to a gene, with labels such as TLR13-1, TLR13-2, and TLR13-3 representing multiple copies or isoforms of *TLR13* for clarity. The accompanying bar plot on the right presents Pearson correlation coefficients for each gene pair, quantifying the strength of their correlation.

The initial results showed that microbial communities differed significantly among the groups (Fig. 5A). At the class level, Alphaproteobacteria exclusively dominated the control samples, with two of the samples not detecting any microbes, while Gammaproteobacteria were greatly enriched in the gills of native deep-sea mussels, representing the mature symbiotic community with the lowest alpha diversity. Interestingly, we found that Gammaproteobacteria were significantly enriched in the gill microbial communities of long-term adapted mussels, leading to a decrease in the proportion of Alphaproteobacteria. Due to the enrichment of Gammaproteobacteria, the alpha diversity index of long-term adapted mussels was higher than that of the samples from the control and native deep-sea mussels (Fig. 5B). We also assessed the composition of microbes in the corresponding environments as a baseline, including seawater and sediment, which were using the publicly available environmental data from the same cold seep (See Methods). The environmental data showed the highest alpha diversity index, with Gammaproteobacteria having lower abundance (~27.77%) in seawater and the lowest abundance (~4.66%) in seabed sediment (Fig. 5A, B). The low abundance of Gammaproteobacteria in the environment confirmed the enrichment of Gammaproteobacteria in the gills of long-term adapted mussels, eliminating the possibility that the results were influenced by contamination in the environment samples. Overall, by comparing the microbial composition and diversity with the environmental samples, we concluded that the microbes in the long-term adapted samples were enriched in the gills rather than originating from the surrounding environment.

Unifrac dissimilarity-based Principal Co-ordinates Analysis (PCoA) analysis of all the samples showed that the microbial communities of long-term adapted mussels have high heterogeneity, appeared to depict the microbial trajectory from control mussels to the native deep-sea mussels. While the environmental samples shown in the lower-right corner (Fig. 5C). We then analyzed the microbial communities at the species level, revealing that approximately 99.88% of *Bathymodiolus platifrons methanotrophic gill symbiont* (NCBI Taxonomy ID: 113268) dominated the gills of native deep-sea mussels (Fig. 5D), indicating a specialized symbiotic community in the cold seep environment as previously reported^39,43^. Interestingly, the gill of long-term adapted mussels showed ~ 26.41% presence of *Bathymodiolus platifrons methanotrophic gill symbiont* (Fig. 5D). Further analysis of each gill sample from long-term adapted mussels unveiled a significant diversity in abundance of this methanotrophic bacteria, varying from 0% to 48.94%, along with the continued presence of the predominant species observed in the control group (Supplementary Fig. 8A). In addition, functional profiles revealed distinct metabolic activities between the LT and Con groups The pathway related to Cofactor, Carrier, and Vitamin Biosynthesis, Fatty Acid and Lipid Biosynthesis, and Carbohydrate Degradation were exclusively enriched in LT group, indicating activated organic metabolism in the microbial communities in the gills of mussels with long-term deep-sea environment exposure (Supplementary Fig. 8B).

We next conducted integrated analyses of the microbial metagenomic data and the transcriptional data of mussels, leveraging the precise one-to-one sample correspondence between transcriptomic changes and the presence of the methanotrophic bacteria, computing the Pearson correlation coefficients between the relative abundance of *Bathymodiolus platifrons methanotrophic gill symbiont* and expression of each gene (Fig. 5E; Supplementary Data 2). The results showed that *TLR* (toll-like receptor) and *MyD88*, involved in the innate immune recognition and the downstream signaling pathway, also in the mediation of endocytosis, were significantly positively correlated with abundance of methanotrophic bacteria (Fig. 5F)^44–46^. Moreover, genes involved in endocytosis, a critical step in establishing the host-symbiont relationship^13^, were also identified (Fig. 5F), including *LRP* (low-density lipoprotein-receptor-related protein), Protocadherin and *VPS* (vacuolar protein-sorting protein). These genes were consistently up-regulated in the long-term samples enriched with methanotrophic bacteria, particularly in LT1 and LT2, which had the highest abundance of methanotrophic bacteria in gills. Interestingly, we noticed that the highly correlated genes were significantly enriched in ribosome and translation (Supplementary Fig. 8C), which had found to be positive correlated with the deep-sea environment using SCOGs in WGCNA analysis (Fig. 4B), may suggest some associations between the activities of ribosome and symbiosis establishing or maintaining.

Overall, from the metagenomic analysis of mussels across diverse locations and conditions, combined with the environmental baseline data, we observed that the microbial community of long-term deep-sea adapted mussels closely resembles that of native deep-sea mussels, displaying a significant enrichment of deep-sea mussel-associated microbes. While no direct evidence of a symbiotic relationship between methanotrophic bacteria and long-term adapted mussels exists, the genes exhibiting a strong positive correlation with methanotrophic bacteria support the previously proposed symbiosis model in deep-sea mussels^13^, which may represent the initial stage in the establishment of a host-symbiont relationship.

**Fig. 6:**
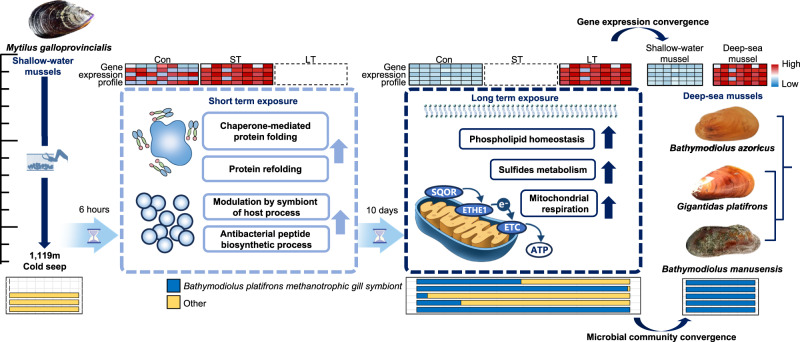
A model of adaptive mechanisms of shallow-water mussels in response to the deep-sea environment. The shallow-water mussels *M. galloprovincialis* were deployed on the seabed of cold seep with 1,119 m, for 6 h (Short term exposure) and 10 days (Long term exposure). Then, mussels were fixed on-site and performed transcriptomic analysis. In short-term exposure, mussels activated mechanisms for homeostasis maintenance and immune response in response to the deep-sea environment. For long-term exposure, mussels enhanced phospholipid homeostasis, and mitochondrial metabolic processes involved in sulfide metabolism and mitochondrial respiration to facilitate adaptation to the deep sea. The left and middle heatmaps revealed the gene expression dynamics for the functions listed below each heatmap across different groups. The right heatmap indicated the upregulated genes found in the long-term exposure mussels were also upregulated on the native deep-sea mussels, compared with the native shallow-water mussels. The relative abundance of methanotrophic bacteria showed convergence to the native microbial community in the gill of deep-sea mussel, during the deep-sea exposure. The image of *M. galloprovincialis* (https://www.mollusca.co.nz/) was adapted with permission. The image of *B. azoricus* was adapted from Wikimedia Commons (https://commons.wikimedia.org/wiki/File:Bathymodiolus_azoricus.jpg), licensed under CC-BY-SA-4.0.

In conclusion, our study provides in situ transcriptomic and metagenomic resources to understand the dynamics of transcriptomic profiles in shallow-water mussels as they adapt to the deep-sea environment, and the microbial community succession during the adaptation. In the early stages, mussels activated mechanisms for homeostasis maintenance and immune response in response to the deep-sea environment (Fig. 6). With prolonged exposure, mussels enhanced mitochondrial metabolic processes involved in sulfide metabolism and mitochondrial respiration to facilitate adaptation to the deep sea. Prolonged exposure to deep-sea also leads to the enrichment of deep-sea specific methanotrophic bacteria, resulting in the activation of immune recognition and endocytosis of the mussels. Confirmed through the convergence of adaptive transcriptional and microbial characteristics between experimental and native deep-sea mussels, along with WGCNA analysis, these findings provide valuable insights into the adaptive evolution of deep-sea mussels within the framework of their radiation evolution.

## Discussion

Evolution and transcriptional regulation are two intertwined aspects of organismal adaptation, operating on vastly different time scales and reversibility^47,48^ Evolution is an irreversible process driven by environmental pressures over millions of years, while transcriptional regulation is reversible and dynamic, enabling immediate responses to current environmental changes within hours or days. The interplay between transcriptional regulation and evolution balances temporary and permanent adaptations and could potentially interact with each other^49^. Studying both rapid transcriptional responses and stable evolutionary adaptations spanning millions of years is crucial for understanding how organisms adapt to their environment and how the environment shapes the course of evolution. However, most studies on deep-sea environmental adaptations focus on the evolution^13,29,50–53^, how organisms directly response and adapt to the deep-sea environment is limited, especially in situ. Here, we examined the transcriptomic shifts in shallow-water mussels *M. galloprovincialis* exposed to deep-sea in situ conditions for 6 h and 10 days (Fig. 1A). We found that mussels survive successfully in the deep sea by regulating their gene expression in a time-dependent manner, with homeostasis and stress responses genes activated first, followed by enhanced metabolism processes of sulfides and energy (Fig. 2C, D). Comparative phylotranscriptomic analysis across its native deep-sea and shallow-water relatives reveal that the deep-sea environment gradually shifts the global transcriptional profile of shallow-water mussels towards that of the native deep-sea mussels (Fig. 3). In addition, improvements in protein biosynthesis and DNA repair are also assist deep-sea mussels in adapting to deep sea (Fig. 4B, C). Importantly, the native symbiont in deep-sea mussels, methanotrophic bacteria, was gradually dominated in the gills of long-term deep-sea mussels (Fig. 5D), genes highly correlated with the abundance of methanotrophic bacteria support the symbiosis model (Fig. 5F). Collectively, these evidences emphasize the role of evolutionarily conserved genes and microbial community shifts in adaptation to extreme environments.

Conducting deep-sea in situ experiments is challenging. Previously, deep-sea adaptability studies have been conducted in the laboratory using pressurized chambers to simulate the high hydrostatic pressure and temperature of deep-sea environments. Pinheiro et al. (2019) used a hyperbaric chamber (4 Bar, 50 Bar) with sediments to pressurize the mussel *M. galloprovincialis*, indicating that high pressure affects glutathione-s-transferase (GST) activity^54^. Liang et al. (2020) incubated the sea cucumber *A. japonicus* under high pressure (15 MPa, 25 MPa), revealed that genes related to ubiquitination, endocytosis, antioxidation, immune response, and DNA repair responded to high-pressure acclimation^17^. Likewise, Chen et al. (2020) investigated the time-series transcriptional response of *A. japonicus* to high-pressure incubations (25 MPa), demonstrating that the dynamic transcriptional response to high pressure was involved in ubiquitination, endocytosis, stress response, methylation regulation, and transmembrane transportation^16^. However, in-lab simulations may not fully replicate the complexity of deep-sea environments, particularly their unique ecosystems. Furthermore, given that transcriptional changes occur rapidly, in situ sample fixation is also necessary to preserve the transcription profile from the moment of collection from the seabed to processing on deck^22,55^. Therefore, to truly understand the ecophysiological processes involved in adapting to the deep-sea environment, we perform in situ adaptation experiments in the deep sea, coupled with in situ fixation of samples (Fig. 1A). To our knowledge, this approach pioneers the integration of in situ experimentation with in situ fixation techniques in the study of deep-sea environmental adaptation.

One of the most interesting results we found is that after the long-term exposure, the expression profiles of shallow-water mussels significantly shifted resemble those of deep-sea mussels, showing a phenomenon of gene expression convergence in response to adaptive shifts. This event of convergent adaption mirrors previous findings in different species, where similar environmental pressures lead to parallel adaptive solutions, including DNA template changes and transcriptome regulation, through independent evolutionary paths^56,57^. For example, in plants, phosphoenolpyruvate carboxylase (PEPC) in C_4_ and Crassulacean acid metabolism (CAM) plants not only shows convergent amino acid substitutions but also higher expression levels^58^. Additionally, in CAM species like *Kalanchoe fedtschenkoi* and *Ananas comosus* (pineapple), 54 genes exhibit shifts in diel gene expression patterns, affecting functions such as nocturnal CO_2_ fixation and circadian rhythm, demonstrating how gene expression convergence can be a key adaptive mechanism^59^. Similarly, study of ray-finned fish species *Fundulus heteroclitus* (mummichog) revealed that global gene expression significantly correlates with habitat temperature across different locations^60^. Overall, all this evidence, combined with our findings, suggests that environmental factors play a critical role in shaping gene expression patterns among organisms adapted to similar environments, resulting in transcriptional convergence in response to adaptive shifts.

Previous DNA-level analysis such as phylogenetics studies comparing the deep-sea mussel *G. platifrons* and the shallow-water mussel *M. philippinarum* has provided insights into their adaptation mechanisms to the deep-sea chemosynthetic environments, and suggests that the deep-sea mussel has largely retained genes from its shallow-water ancestors^13^. On the other hand, comparative transcriptomic analyses between native shallow-water and deep-sea mussels have shown conserved gene families related to stress responses, without any specifically expanded families, while gene expressions associated with symbiosis exhibit significant variation^3^. Building upon these findings, our study narrows the focus to the gene expression changes within the same species, *G. platifrons*, to characterize the transcriptional adaption to the deep-sea environment, and reveal a time-dependent response to the deep-sea environment. Initially, mussels trigger pathways crucial for maintaining homeostasis and managing stress. Over the long term, they shift towards pathways that facilitate metabolic adjustments and enhancements (Figs. 2C, 3D; Supplementary Fig. 3B). Such a time-dependent response has been observed in the sea cucumber *A. japonicus*, where exposure to high pressure led to increased expression of stress response and ubiquitination genes at 6 h, followed by the activation of methylation regulation and transmembrane transportation at 24 h. Homeostasis maintenance and immune response are among the first biological processes to be activated when mussels are exposed to the deep-sea environment (Fig. 2C). Within 6 h of exposure, genes associated with protein and phospholipid homeostasis are rapidly upregulated to stabilize protein structures^61^ and maintain the integrity of cellular membranes, which are highly sensitive to the effects of high hydrostatic pressure^62,63^. Concurrently, immune and stress-related genes exhibit a similar trend of rapid activation. Several studies reveal the immune and stress response genes were upregulated under the pressure environment. For example, high hydrostatic pressure activates immune and stress responses in sea cucumber *A. japonicus*^17^ and shallow-water amphipod *Eogammarus possjeticus*^15^. High-density rearing initiates the immune and inflammatory responses in European seabass (*Dicentrarchus labrax*)^64^. Additionally, hydrostatic pressure could also lead to the suppression of microtubule cytoskeleton organization and cilium movement, as evidenced by both our results (Fig. 3D) and the findings of Sun et al. ^13^, which show that cytoskeleton-related genes are highly expressed in shallow-water mussels but not in their deep-sea counterparts.

Metabolic adaptation emerges as a critical mechanism driving mussel survival in the deep sea, with enhanced sulfide and energy metabolism in the mitochondria being activated 10 days after exposure to the deep-sea environment (Fig. 2C). The deep-sea chemosynthetic environments are rich in reducing substances such as methane and sulfides^65^, and excessively high levels of hydrogen sulfide can be toxic to organisms^66^. Our analyses revealed the activation of pathways from sulfide oxidation to ATP metabolic processes in the long term (Fig. 2C), highlighting the crucial role of sulfide metabolism in the adaptation of mussels to the deep sea. The identification of hub genes, including NADH dehydrogenases, COX11 and TPMT (Fig. 4C), further emphasizes the importance of efficient energy metabolism and detoxification processes^67^. The upregulation of genes related to sulfide oxidation and the mitochondrial respiratory chain serves as a detoxification response to the high sulfide levels in the deep sea, aligning with findings from transcriptomic analyses of deep-sea mussels in hydrothermal vents and methane seep areas, as well as experiments involving Na_2_S exposure^21^. In addition, the high similarity of transcription profile between our long-term deep-sea exposure mussels and native deep-sea mussels confirmed that specialized metabolism process is a decisive factor in the adaptation of mussels to the extreme deep-sea environment (Figs. 3E–G, 4A, Supplementary Fig. 5).

Deep-sea mussels sustain themselves in extreme environments through symbiosis with sulfur-oxidizing and methanotrophic bacteria, which utilize sulfide and methane as energy sources. These bacteria, acquired through horizontal transmission, are phagocytosed and housed in gill epithelial cells to establish a symbiotic relationship^37^. Mussel gill filters seawater to capture the particle, the microbes on the seawater carry signature molecules on their surface, which can be recognized by the transmembrane pattern-recognition receptors (PRRs) of the mussel^68^. Our data revealed that the genes involved in innate immune recognition and endocytosis were highly correlated with the abundance of methanotrophic bacteria in the gills of long-term adapted mussels (Fig. 5F). These genes have been reported in playing roles symbiotic establishment^13^. Peptidoglycan recognition proteins and TLR in gill cells serve as the recognition system for gamma-proteobacteria, potentially triggering the process of phagocytosis^69,70^. In mammalian microglial cells, which play critical surveillance and phagocytic roles in the brain, TLR activity enhances phagocytosis through the MyD88 signaling cascade^71^. This TLR-MyD88 signaling pathway closely correlates with the abundance of methanotrophic bacteria in our data (Fig. 5F). Tame et al. ^72^ demonstrate that the gill epithelial cells of deep-sea mussels non-selectively phagocytose exogenous bacteria and enclose them in phagosomes, and can selectively manipulate the retention of symbionts and eliminate other exogenous bacteria during phagosome digestion after engulfment. In essence, the immunological system and phagocytosis mediate the symbionts recognition and retention. Moreover, in invertebrates, which lack adaptive immunity, the effective and complex innate immune system not only defends against external pathogens, but also plays important roles in interacting with symbionts^73^. Previous studies have shown that the reduction of immune response genes facilitates the host-symbiont relationship^3,74,75^. The enrichment of methanotrophic bacteria, the short-term activation and long-term inactivation of immune-related genes suggest an initial response to environmental microbial invasion, followed by adaptation to the surrounding microbial environment. In addition, that local translation within microglial cells supports efficient phagocytosis, with local protein synthesis aiding in the effective handling of pathogen-like particles^76^, this may support evidence about the activities of translation and ribosome assembly were highly correlated with methanotrophic bacteria (Supplementary Fig. 8C). Our findings align closely with the native mechanisms of deep-sea mussels in forming symbiotic relationships. It is certain that further investigation is necessary to elucidate the detailed mechanisms behind the specific enrichment of deep-sea symbionts, such as specific preference for methanotrophic bacteria as symbionts by mussels. In general, our integrated results highlight the roles of immune regulation, endocytosis, and ribosome activity in this enrichment and in establishing the symbiotic relationship.

Overall, this study highlights the complex transcriptional responses interplay between homeostasis, immune and metabolisms, illustrating how this relationship is instrumental in supporting the adaptation of experimental mussels to the deep-sea environment. It additionally suggests the critical role of sulfide metabolism within the distinct chemosynthetic environments of the deep sea. Over time, these mussels have shown an enhanced capacity for nutrition metabolism, evidenced by their participation in diverse organic metabolic activities (Supplementary Fig. 2). Prior research has identified symbionts as crucial allies for deep-sea mussels, providing not only organic carbon and other vital nutrients but also alleviating the impact of toxic substances, thus protecting the mussels^77–79^. This evidence suggests the importance of symbiotic relationships, potentially enabling shallow-water mussels to access the resources and energy needed to prosper in chemosynthetic ecosystems over evolutionary time. The microbial communities in gills of shallow-water mussel experienced complex succession, as the native symbiont in deep-sea mussel gill, methanotrophic bacteria gradually emerged as a dominant group in the long-term adapted mussel. The potentially highly correlated genes support the symbiosis model of the deep-sea mussel in transcriptional level. However, our study lacks longer periods data to examine the interaction between mussel host and bacteria, an area for potential future enhancement.

## Methods

### Samples Collection

In May 2021, the shallow-water mussel *M. galloprovincialis* was harvested from the intertidal zone in Qingdao, China. These mussels were subsequently transported in coolers to the laboratory, where they underwent a month-long acclimatization process in a closed recirculating aquaculture system. This acclimatization aimed to adapt them to laboratory conditions and mitigate the stress from their collection and handling. Throughout this period, the mussels were kept in sand-filtered seawater at a salinity of 34 ± 1‰, under a natural light cycle, and at a chilly temperature of 4°C. No food was provided during acclimatization, and the seawater was partially renewed weekly. At the end of this period, five mussels were dissected, and their gills were immediately preserved with RNAlater (Omega Biotek, USA), and flash-frozen with liquid nitrogen after being kept overnight at 4°C, thereby establishing the control group for the study.

The in situ experiment was carried out at Site-F cold seep of South China sea at a depth of 1119 m, where the deep-sea mussel *G. platifrons* predominates as the major macrobenthic organism. Twenty shallow-water mussels were taken down to the seabed in a plastic box filled with ice, using the remotely operated vehicle (ROV) *Faxian*, deployed from the research vessel *Kexue*. To mitigate the potential of displacement by ocean currents, the container was weighted at its base. During the experiment, the container’s lid was kept closed to prevent the mussels from dispersing into the surrounding environment. Perforations in the container facilitated continuous water exchange with the ambient seawater, ensuring the mussels were exposed to the deep-sea in situ conditions. Following exposure periods of 6 h (short-term pressure: ST) and 10 days (long-term pressure: LT) to the ambient deep-sea conditions, 10 mussels were fixed using a custom-designed High-throughput in situ fixation device designed for the preservation of deep-sea macroorganisms’ RNA (Fig. 1A). This device comprised four in situ fixation buckets (5 L each) and a movable frame that can be mounted on the ROV. The in situ fixation buckets were pre-filled with in-house RNA stabilizing solution on board before being lowered to the underwater experimental site by ROV *Faxian*. The bucket was covered in the inside by a sealing rubber to avoid seawater coming into contact with the RNA stabilizing solution during the sample fixation. Prior to their placement in the fixation device, the shallow-water mussels were gently cracked using the manipulator arm of the ROV. Following the fixation, the buckets were securely sealed by closing the lid and were then returned to the ROV’s sample basket. Upon retrieval of the ROV onboard, the sampling buckets were immediately transported to the onboard laboratory. The remaining non-fixed mussels were transported onboard using insulated device. The on-site fixed mussels were immediately dissected to harvest gill tissues, which were then preserved with RNAlater (Omega Biotek, USA) and subjected to flash-freezing in liquid nitrogen following an overnight stabilization at 4°C. The preserved specimens were stored at -80°C until further analysis. The RNA stabilizing solution was prepared according to the specifications outlined by Mat et al. (2020)^80^ and was maintained at 4°C in the ship’s cold room prior to the ROV deployment.

### Sequencing and assembly of the reference transcriptome

Total RNA from gill tissues was extracted using TRIzol and sent to Novogene Bioinformatics Technology Co., Ltd (Beijing, China) for sequencing. Eukaryotic mRNA clusters were generated, and the library preparations were sequenced on the Illumina Novaseq 6000 platform, generating 150 bp paired-end reads. Raw reads were conducted quality control by fastp version 0.12.4^81^ with default parameters.

We attempted to align the reads to the reference genome of *M. galloprovincialis* (Genbank accession number: GCA_025277285.1). However, due to the low mapping rate (~ 59.41%), we conducted de novo assembly of the reference transcriptome using Trinity version 2.13.2^82^ with default parameters, except for both “--min_kmer_cov” and “--min_glue” of 3 options to remove the low abundance transcripts. The longest transcript of each gene was extracted using the script “get_longest_isoform_seq_per_trinity_gene.pl” affiliated with Trinity. These transcripts were then clustered to elimination redundancy using CD-HIT version 4.8.1^83^ with a sequence identity threshold of 0.9, resulting in the generation of the reference transcriptome, named unigenes. The completeness of unigenes was assessed using Benchmarking Universal Single-Copy Orthologs (BUSCO) version 5.3.2^84^ against eukaryota_odb10 database.

### Transcriptome functional annotation

We annotated putative functional characteristics of unigenes following the Trinotate protocol^85^. Diamond blastx version 2.0.14.152^86^ was utilized to search unigenes against the Non-Redundant Protein Sequence Database (NR) with a cutoff E value of 1e-5. Subsequently, TransDecoder version 5.5.0 (github.com/TransDecoder/TransDecoder) predicted the likely coding regions within unigenes, and the sequences of open reading frame (ORFs) were used BLASTp version 2.12.0^87^ to search against comprehensive protein database by merging Swiss-Prot^88^ with UniRef90 database^89^, with a cutoff E value of 1e-5. The conserved protein domains of ORFs were searched using hmmscan version 3.1b2^90^ against the Pfam database^91^. All functional annotations were parsed by Trinotate, which then generated the Gene Ontology (GO)^92^ annotations of unigenes.

### Transcripts abundance estimation, differential expression analysis and function enrichment

The transcriptome profiles were processed as follows: firstly, clean data were aligned to unigenes using bowtie version 1.3.1^93^. Next, abundance estimation was quantified by Salmon version 1.7.0^94^, and the genes expression levels were measured in transcripts per million (TPM). DESeq2 R package version 1.38.3^95^ was employed to identify genes with significantly different expressions between groups, with genes having |log2FoldChange| > 1 and p-adjust < 0.05 defined as differentially expressed genes (DEGs).

For GO enrichment analysis and visualization, clusterProfiler R package version 4.6.2^96^ was used in conjunction with the ggplot2 R package version 3.4.2^97^. GO terms with a *p*-value < 0.05 were considered as the significantly enriched. Terms were condensed using REVIGO^98^. The alluvial plot was generated using the ggalluvia R package version 0.10.0^99^.

### Orthology inference and phylotranscriptomic analysis

To perform the phylotranscriptomic analysis, we obtained 17 publicly available RNA-Seq samples from 3 deep-sea species: *B. azoricus*^80^, *B. manusensis*^3^, *G. platifrons* (Basionym: *B. platifrons* Hashimoto & Okutani, 1994)^3^, and 3 shallow-water species: *M. coruscus*^100^, *M. galloprovincialis*^101^, *P. viridis*^102^,which were downloaded from NCBI SRA. The metadata of the public data with SRA accession numbers were detailed on Supplementary Table 3. We conducted quality control of raw reads, de novo assembly, BUSCO assessment, functional annotation, reads alignment, and abundance estimation with the same processing parameters as for our experimental mussels unigenes.

OrthoFinder version 2.5.4^27^ was used to infer the phylogenetic relationships among all seven species using ORFs sequences. The identified single-copy orthologous genes (SCOGs) were used to infer the phylogenetic tree with the “-M msa” option, and the tree was visualized using the ggtree R package version 3.6.2^103^.

### Single-copy orthologous genes differentially expressed analysis

To reveal the transcriptional dynamics of SCOGs among mussels in our experiment and native species, we integrated expression data from Con, LT and all public samples. We then corrected for batch effects using the ‘ComBat_seq’ function from the sva R package version 3.35.2^104^, which utilizes the raw count matrix as input. Subsequently, the corrected count matrix was input into DESeq2. To reduce the number of false positive candidate genes in the differentially expressed analysis^105^, we constructed a transcripts length matrix with row-wise geometric means of 1, which was applied using the ‘normalizationFactors’ function in the DESeq2 R package. This normalization factor matrix includes gene length to account for the length differences between orthologous genes among all unigenes. To accurately evaluate the transcriptional correlation between the native comparison (Deep vs Shallow) and experimental comparison (LT vs Con), we performed differential expression analysis to identify native species differential expression SCOGs (DESCOGs) with the same criteria as for DEGs. We also re-identified the DESCOGs between the LT and Con. All correlation analyses were conducted using ‘cor.test’ function in stats R package version 4.2.2.

### Co-expression analysis

The corrected SCOGs count matrix of all species was used as input for Weighted Gene Co-expression Network Analysis (WGCNA) to construct a scale-free co-expression network^28^. Modules with a minimum of 30 members and those below 0.3 in dynamic clustering tree were then merged. Module with a p-value < 0.05 in relation to the sample traits was considered as interesting candidate. The ‘GSEA’ function within the clusterProfiler R package was used to explore the core functions of candidate module. The network visualization of module was presented using Cytoscape version 3.9.1^106^. cytoHubba was used to identify the hub genes^107^.

### Positive selected gene identification

To identified positively selected genes (PSGs), the SCOGs were aligned using ParaAT version 2.0 (ngdc.cncb.ac.cn/tools/paraat) with the “-g” and “-m mafft” options. We then conducted optimized CallCodeml (github.com/byemaxx/callCodeml) to call CODEML of the phylogenetic analysis by maximum likelihood (PAML) package version 4.9j^108^ in bulk to calculate the positive selection using the branch-site model. The phylogenetic tree branch containing all deep-sea species was defined as the ‘foreground’ phylogeny for calculating the selection pressure, labeled with “$1” on the tree. Chi-squared test was used to test the likelihood value between the alternative model (model = 2, NSsites = 2, fix_omega = 0, omega = 2) and null hypothesis (model = 2, NSsites = 2, fix_omega = 1, omega = 1). SCOGs with a p-value < 0.05 and Bayesian Empirical Bayes (BEB) sites > 0.9 were considered as PSGs.

### Shotgun metagenomic DNA extraction and sequencing

To examine the dynamics of microbial communities in gills of mussels in control and long-term adapted state, a total of 0.2 μg DNA per sample was used as input material for the DNA library preparations. Initially, the genomic DNA sample was fragmented by sonication to reach a size of 350 bp. Next, the DNA fragments underwent end polishing, A-tailing, and ligation with the full-length adapter for sequencing, followed by PCR amplification. The PCR products were then purified using the AMPure XP system (Beverly, USA). Subsequently, the library quality was assessed on the Agilent 5400 system (Agilent, USA) and quantified using QPCR (1.5 nM). After conducting library quality control, the different libraries were pooled based on their effective concentration and the desired data amount. The 5’-end of each library was phosphorylated and cyclized. Then, loop amplification was performed to generate DNA nanoballs. Finally, these DNA nanoballs were loaded into a flow cell with DNBSEQ-T7 for sequencing at Novogene Bioinformatics Technology Co., Ltd (Beijing, China).

### Metagenomic analysis

To compare the microbial compositions among experimental mussel gills, native deep-sea mussel gill, and surrounding environments, we obtained 19 publicly available shotgun metagenomic samples, including 11 native deep-sea mussel gill (*G. platifrons*), 4 seawater and 3 seabed sediment samples (Supplementary Table 4). For better comparability, all public data were sampled from Site-F cold seep.

All raw reads were conducted quality control by fastp version 0.12.4^81^ with default parameters. Our clean experimental metagenomic data were aligned to unigenes of mussels using the built-in bowtie2 version 2.4.2^109^ of KneadData version 0.12.0 (github.com/biobakery/kneaddata). The native deep-sea mussel gills’ data were filtered against with *G. platifrons* genome (GenBank accession no. JAOEFJ000000000). Then, all metagenomic data were analyzed the taxonomic composition by MetaPhlan version 4.1.1^110^. Functional annotations of MetaCyc pathways abundances were performed by HUMAnN version 3.9^111^, with “--prescreen-threshold” of 0.000001 option.

For analysis and visualization of the metagenomic data, the merged species table with abundance were imported into phyloseq R package version 1.46.0^112^. The visualization of metagenomic interindividual variation by PCoA was based on Unifrac dissimilarity matrix. In order to corresponding with Unifrac dissimilarity. We generated the relative average abundance of each group with hierarchical clustering also based on Unifrac dissimilarity and “average” of hcluter_method parameter, by the optimized “tax_stack_clust” function of amplicon R package version 1.19.0^113^.

To find the highly correlated genes in response to *Bathymodiolus platifrons methanotrophic gill symbiont*, we computed the Pearson correlation coefficient between the expressions and the abundance of *Bathymodiolus platifrons methanotrophic gill symbiont* in all Con and LT samples. The abundance of *Bathymodiolus platifrons methanotrophic gill symbiont* were firstly normalized based upon quantiles^114^ using preprocessCore R package version 1.64.0 (github.com/bmbolstad/preprocessCore). The differently abundant pathway analysis was analyzed using “trans_diff” function of “microeco” R package v1.4.0^115^. The Wilcoxon Rank Sum test was employed to assess the significance of differences in pathway abundances. “cor” and “corPvalueStudent” functions of WGCNA were conduct to rapidly compute Pearson correlations and Student asymptotic p-values. Genes with correlation > 0.65 were further performed the GO enrichment analysis.

### Statistics and reproducibility

A total of 5 biological replicates of mussels per group were used for transcriptomic and metagenomic sequencing in this study. The sample sizes for transcriptomic and metagenomic datasets obtained from publicly available sources are detailed in Supplementary Table 3 and Supplementary Table 4, respectively. Unless otherwise specified above or in the figure legends, data are presented as the median or mean ± SEM. The Wilcoxon Rank Sum test was employed to perform statistical tests between groups. The level of significance was determined at *p*  <  0.05. All statistical analyses and data visualizations were performed using R (version 4.3.1; R Core Team), unless otherwise specified.

### Reporting summary

Further information on research design is available in the Nature Portfolio Reporting Summary linked to this article.

## Supplementary information

Supplementary Information

Description of Additional Supplementary Materials

Supplementary Data 1

Supplementary Data 2

Reporting summary

## Data Availability

Raw RNA-seq sequencing data, de novo assembly, and RNA-Seq expression matrix in this study have been deposited in NCBI’s Gene Expression Omnibus (GEO) and are accessible through GEO series accession number GSE263620. Shotgun metagenomic data generated in this study have been deposited in NCBI’s Sequence Read Archive (SRA) with run accessions: SRR29152236 - SRR29152234. All the repositories of publicly available RNA-seq and metagenomic data used in this study are detailed in Supplementary Table 3 and Supplementary Table 4. The source data behind the graphs are available at Figshare (10.6084/m9.figshare.27875763.v2). All other data are available from the corresponding author on reasonable request.

## Code availability

Codes used in this study are available in GitHub (https://github.com/XiaoluLLLLLiu/Xiaolu2024CommunicationsBiology) and Zenodo (https://zenodo.org/records/14271388)^116^. Additional analytical scripts can be obtained from the corresponding author upon reasonable request.
