# Supplementary material for: Shallow-water mussels (Mytilus galloprovincialis) adapt to deep-sea environment through transcriptomic and metagenomic insights
Source: Commun Biol. 2025 Jan 14;8:46. doi: 10.1038/s42003-024-07382-0 (PMC11729891; doi:10.1038/s42003-024-07382-0)
Supplement: Supplementary file 1 — Supplementary Information [file 42003_2024_7382_MOESM1_ESM.pdf]

SUPPLEMENTARY INFORMATION

Shallow-water Mussels (*Mytilus galloprovincialis*) Adapt to Deep-Sea Environment Through Transcriptomic and Metagenomic Insights

Luyang Sun[1,2,6 # *], Xiaolu Liu[1,2,6 #], Li Zhou[3,6 #], Hao Wang[3,6], Chao Lian[3,6], Zhaoshan Zhong[3,6], Minxiao Wang[3,6], Hao Chen[3,6], Chaolun Li[3,4,5,6 *]

[1] Single-Cell Center, CAS Key Laboratory of Biofuels, Shandong Key Laboratory of Energy Genetics, Shandong Energy Institute, Qingdao New Energy Shandong Laboratory, Qingdao Institute of Bioenergy and Bioprocess Technology, Chinese Academy of Sciences, Qingdao 266101, China

[2] Laboratory for Marine Biology and Biotechnology, Qingdao Marine Science and Technology Center, Qingdao 266104, China

[3] Center of Deep Sea Research, Institute of Oceanology, Chinese Academy of Sciences, Qingdao 266071, China

[4] South China Sea Institute of Oceanology, Chinese Academy of Sciences, Guangzhou 510301, China

[5] CAS Key Laboratory of Marine Ecology and Environmental Sciences, Institute of Oceanology, Chinese Academy of Sciences, Qingdao 266071, China

[6] University of Chinese Academy of Sciences, Beijing 10049, China

[*] **Corresponding author:**

Dr. Luyang Sun, E-mail: sunly@qibebt.ac.cn. Qingdao Institute of Bioenergy and Bioprocess Technology, Chinese Academy of Sciences, Qingdao 266101, China

Dr. Chaolun Li, E-mail: lcl@qdio.ac.cn. South China Sea Institute of Oceanology, Chinese Academy of Sciences, Guangzhou 510301, China

[#] **These authors contribute equally to this work.**

TABLE OF CONTENTS

Supplementary Fig. 1: *In situ* exposure and sampling strategy performed on cold seep seabed.

Supplementary Fig. 2: Completeness assessment and functional annotation statistics.

Supplementary Fig. 3: Evidence of gene expressions and GO enrichment results in supporting the survival status of mussels on the deep-sea environment in long-term.

Supplementary Fig. 4: Heat map of DEGs involved in sulfides metabolism.

Supplementary Fig. 5: PCA results from the exploration of batch effect correction.

Supplementary Fig. 6: Scatter plot of the shared DESCOGs with correlation analysis.

Supplementary Fig. 7 Co-expression network construction.

Supplementary Fig. 8 Integrated analyses between microbial and transcriptional data

Supplementary Table. 1 Summary of de novo assembled unigenes

Supplementary Table. 2 Mapping rates of samples aligned with unigenes

Supplementary Table. 3 Metadata and assembled unigenes of shallow- and deep-sea species from SRA

Supplementary Table. 4 Metadata of publicly available shotgun metagenomic samples from SRA

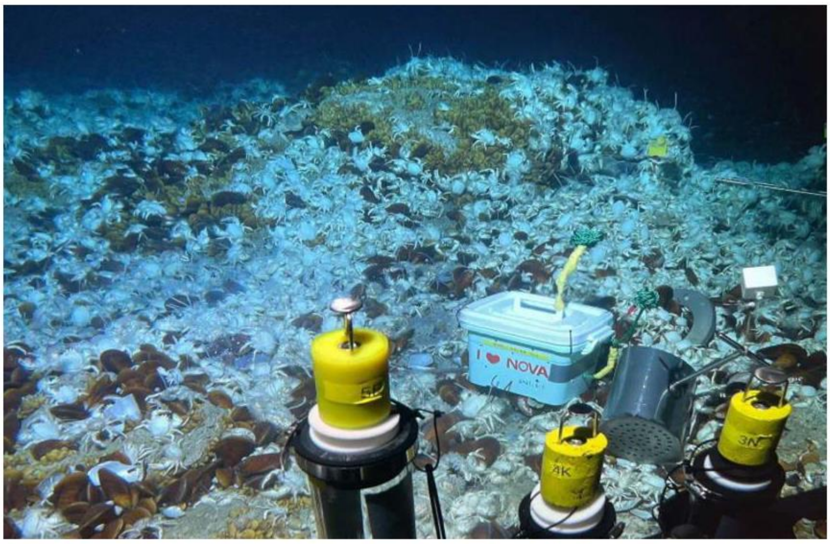

Supplementary Fig. 1: *In situ* exposure and sampling strategy performed on cold seep seabed. The *in situ* experiment was carried out at Site-F cold seep of South China sea at a depth of 1,119 m. The shallow-water mussels were taken down to the seabed in a plastic box filled with ice, using the remotely operated vehicle (ROV) *Faxian*, deployed from the research vessel *Kexue*. Following exposure, the mussels were fixed using a custom-designed High-throughput *in situ* fixation device.

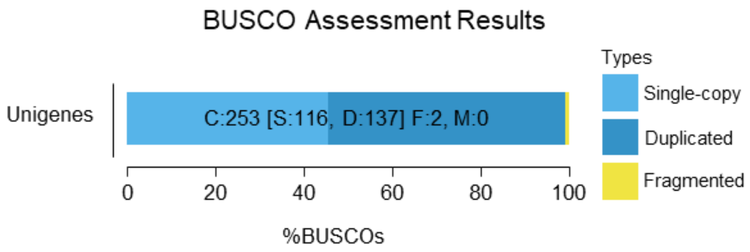

Supplementary Fig. 2: Completeness assessment and functional annotation statistics.

This figure presents the results of unigene completeness assessed using BUSCO.

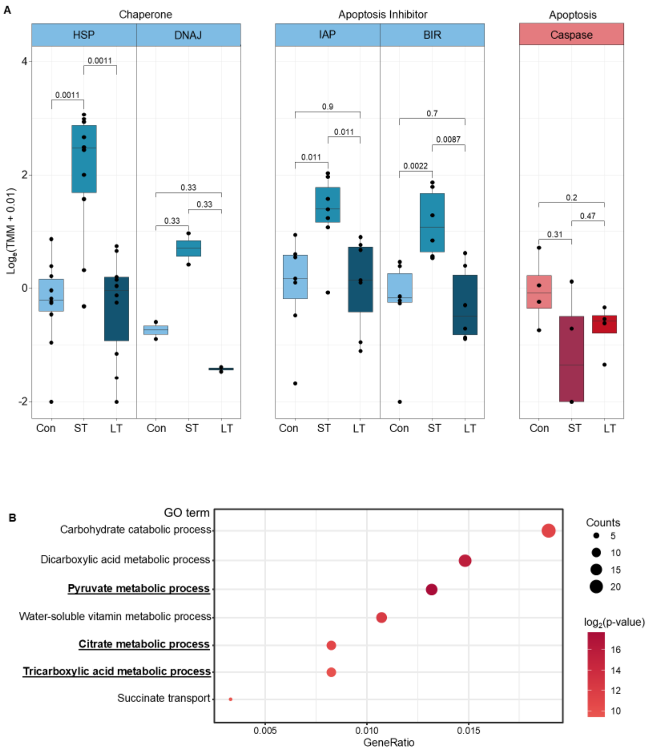

Supplementary Fig. 3: Evidence of gene expressions and GO enrichment results in supporting the survival status of mussels on the deep-sea environment in long-term.

A. Boxplots showing gene expression levels among three groups. Genes involved in chaperone, apoptosis inhibitor, and caspase were plotted using DEGs expressions between ST and Con. Group comparisons were tested using Wilcoxon rank sum test. HSP, heat shock protein; IAP, inhibitor of apoptosis protein; BIR, baculoviral IAP repeat. **B**. GO biological process enrichment analysis used the up-regulated DEGs between LT and Con.

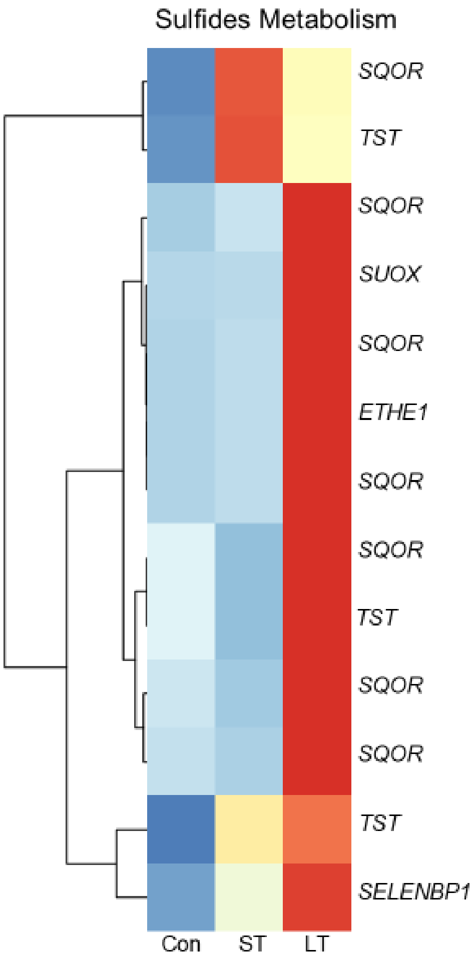

Supplementary Fig. 4: Heat map of DEGs involved in sulfides metabolism.

Heat map depicting the DEGs involved in sulfides metabolism among the groups, with a predominance of up-regulation in the long-term group, suggesting that the activated mechanism of sulfides metabolism played a significant role in response to the deep-sea environment for mussels. *SQOR*: Sulfide Quinone Oxidoreductase; *TST*: Thiosulfate Sulfurtransferase; *SUOX*: Sulfite Oxidase; *SELENBP1*: Selenium Binding Protein 1.

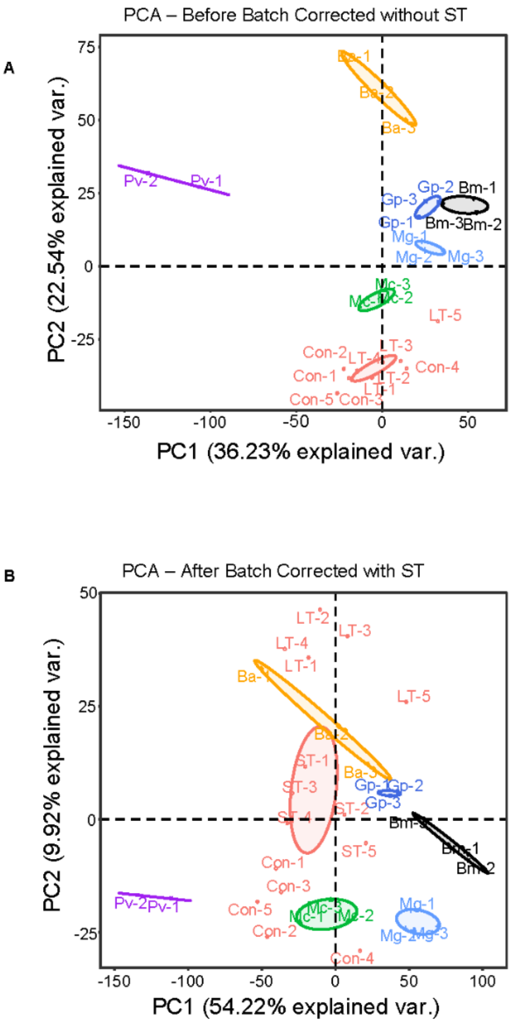

Supplementary Fig. 5: PCA results from the exploration of batch effect correction.

The exploration of PCA using the integrated expression matrix. In Fig. 3E, to obtain a clear result in exploring the dynamic patterns between our long-term experiments and native species, we used the all of sample profiles excepted for the ST group. **A.** PCA result from the integrated expression matrix excluding the ST group, before the batch effect correction. **B**. PCA result from the integrated expression matrix included ST group, after the batch effect correction.

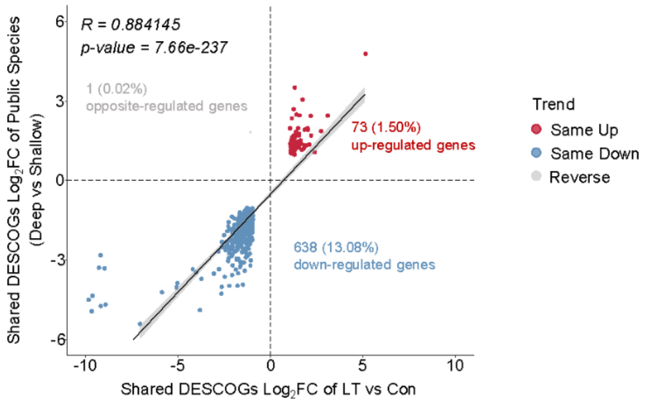

Supplementary Fig. 6: Scatter plot of the shared DESCOGs with correlation analysis.

The scatter plot displays the Pearson Correlation Coefficient of $\log_2$FoldChange for shared DESCOGs between LT vs. Con and Deep vs. Shallow.

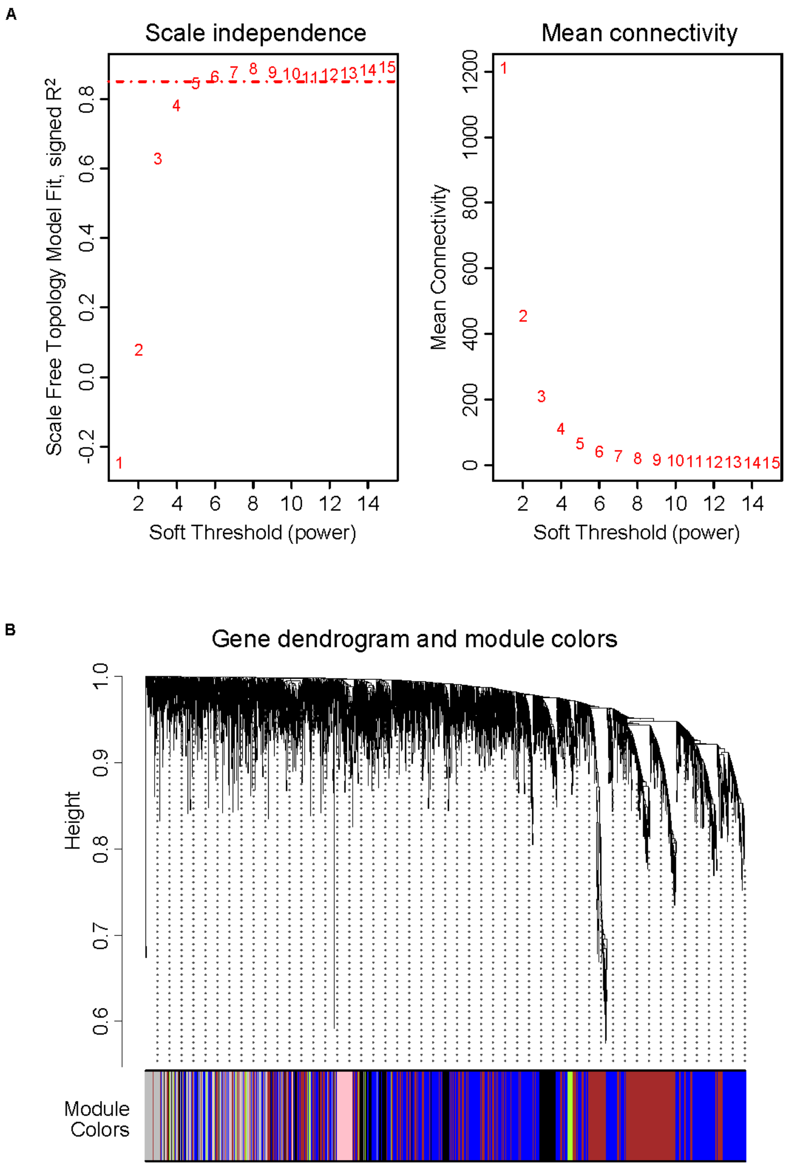

Supplementary Fig. 7 Co-expression network construction.

A. To select the optimal soft threshold for constructing the scale-free network, we used the ‘pickSoftThreshold’ function in WGCAN R package to calculate R-square (left) to evaluate the fit of the scale-free topology model. The filtering criterion was set as R-square = 0.85, leading to the selection of 6 as the soft threshold. The mean connectivity of each soft threshold in the scale-free topology was calculated (right). **B.** Clustering dendrogram of all SCOGs with dissimilarity based on topological overlap. Modules are represented by colors; after merging modules with the dissimilarity below 0.3, six modules were identified.

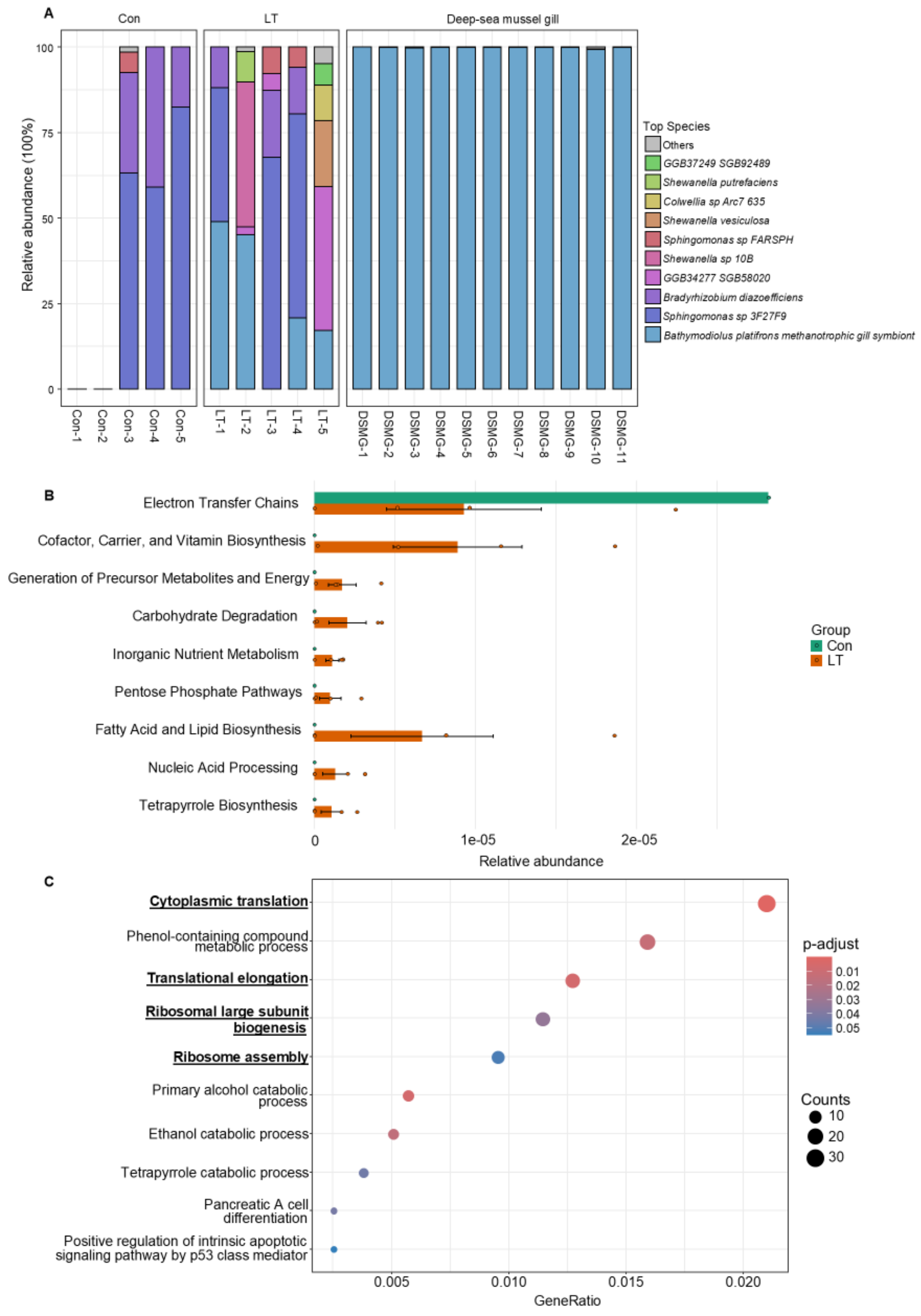

Supplementary Fig. 8 Integrated analyses between microbial and transcriptional data

A. Taxonomic composition in relative abundances of the top 10 species among the experimental and public data. DMSG, deep-sea mussel gill. **B**. The bar plot illustrates the functional profiles of metagenomics, highlighting the differences in MetaCyc pathways between the LT and Con groups; the top 9 pathways are displayed based on Wilcoxon Rank Sum test statistical analysis results. **C**. GO enrichment analysis using the genes which highly correlated with the abundance of *Bathymodiolus platifrons methanotrophic gill symbiont*.

Supplementary Table. 1 Summary of *de novo* assembled unigenes

	Mean length	Max length	N50	Ex90N50	Total reads
Unigenes	581.6	62,228	772	1,780	495,264

Supplementary Table. 2 Mapping rates of samples aligned with unigenes

Sample ID	Con-1	Con-2	Con-3	Con-4	Con-5	ST-1	ST-2	ST-3	ST-4	ST-5	LT-1	LT-2	LT-3	LT-4	LT-5
Mapping Rate	85.17%	87.04%	85.03%	88.08%	85.72%	86.43%	86.60%	86.06%	85.05%	85.79%	86.41%	86.33%	86.91%	86.40%	89.17%

Supplementary Table. 3 Metadata and assembled unigenes of shallow- and deep-sea species from SRA

Environment	Run	BioProject	Instrument	Organism	Unigenes stats	DOI
Deep	ERR3600856<br>ERR3600859<br>ERR3600860	PRJEB34925	Illumina NovaSeq 6000	*Bathymodiolus azoricus*	103,883	10.1038/s41467-020-17284-4
	SRR5164454<br>SRR5164455<br>SRR5168014	PRJNA360359	Illumina HiSeq 2500	*Bathymodiolus manusensis*	107,023	10.1111/mec.14160
	SRR5164456<br>SRR5164457<br>SRR5164458	PRJNA360359	Illumina HiSeq 2500	*Gigantidas platifrons*	146,292	10.1111/mec.14160
Shallow	SRR18682404<br>SRR18682405<br>SRR18682412	PRJNA824625	Illumina HiSeq 2500	*Mytilus galloprovincialis*	105,254	10.21203/rs.3.rs-1536907/v1
	SRR16293785<br>SRR16293786<br>SRR16293787	PRJNA770460	Illumina NovaSeq 6000	*Mytilus coruscus*	112,657	10.3389/fmars.2022.1014336
	SRR1503507<br>SRR1503508	PRJNA360359	Illumina Genome Analyzer IIx	*Perna viridis*	111,568	10.1186/1471-2164-15-804

Table.S4 Metadata of publicly available shotgun metagenomic samples from SRA

Environment	SampleID	Run	BioProject	Instrument	Description	DOI
Seawater	Seawater-1	SRR13892591	PRJNA707313	Illumina HiSeq X Ten	Fluid under the invertebrate communities	10.1038/s41597-022-01586-x
	Seawater-2	SRR13892595			Cold seep fluids at gas plume	
	Seawater-3	SRR13892606			Water closely above the invertebrate communities_2	
	Seawater-4	SRR13892607			Water closely above the invertebrate communities_1	
Surface sediment	Sediment-1	SRR11306546	PRJNA612576	Illumina HiSeq 2500	Methane cold seep marine sediment 0-0.02 cmbsf	10.1016/j.marpolbul.2022.113458
	Sediment-2	SRR13892590	PRJNA707313	Illumina HiSeq X Ten	Sediment (0-2 cmbsf)	10.1038/s41597-022-01586-x
	Sediment-3	SRR19020598	PRJNA831433	Illumina NovaSeq 6000	Methane cold seep marine sediment 0-0.04 cmbsf	10.1038/s41467-023-36877-3
Deep-sea mussel gill	DMSG-1	SRR21936140	PRJNA891060	Illumina HiSeq X Ten	Site-F Methane cold seep mussel gill	10.1186/s40168-023-01695-8
	DMSG-2	SRR21936139				
	DMSG-3	SRR21936128				
	DMSG-4	SRR21936125				
	DMSG-5	SRR21936124				
	DMSG-6	SRR21936123				
	DMSG-7	SRR21936122				
	DMSG-8	SRR21936121				
	DMSG-9	SRR21936120				
	DMSG-10	SRR21936119				
	DMSG-11	SRR21936138				
