# Supplementary material for: Shallow-water mussels (Mytilus galloprovincialis) adapt to deep-sea environment through transcriptomic and metagenomic insights
Source: Commun Biol. 2025 Jan 14;8:46. doi: 10.1038/s42003-024-07382-0 (PMC11729891; doi:10.1038/s42003-024-07382-0)
Supplement: Supplementary file 2 — Description of Additional Supplementary Materials [file 42003_2024_7382_MOESM2_ESM.pdf]

Description of Additional Supplementary Files

File name: Supplementary Data 1

Description: The source data of positively selected genes mentioned in Fig. 4 B and C and Line 380- 381.

File name: Supplementary Data 2

Description: The source data behind the Fig. 5F in the paper.
