# Supplementary material for: Shallow-water mussels (Mytilus galloprovincialis) adapt to deep-sea environment through transcriptomic and metagenomic insights
Source: Commun Biol. 2025 Jan 14;8:46. doi: 10.1038/s42003-024-07382-0 (PMC11729891; doi:10.1038/s42003-024-07382-0)
Supplement: Supplementary file 5 — Reporting summary [file 42003_2024_7382_MOESM5_ESM.pdf]

nature portfolio

Corresponding author(s): Luyang Sun

Last updated by author(s): Dec 2, 2024

Reporting Summary

Nature Portfolio wishes to improve the reproducibility of the work that we publish. This form provides structure for consistency and transparency in reporting. For further information on Nature Portfolio policies, see our Editorial Policies and the Editorial Policy Checklist.

Statistics

For all statistical analyses, confirm that the following items are present in the figure legend, table legend, main text, or Methods section.

n/a	Confirmed	
☐	☒	The exact sample size (*n*) for each experimental group/condition, given as a discrete number and unit of measurement
☐	☒	A statement on whether measurements were taken from distinct samples or whether the same sample was measured repeatedly
☐	☒	The statistical test(s) used AND whether they are one- or two-sided<br>*Only common tests should be described solely by name; describe more complex techniques in the Methods section.*
☒	☐	A description of all covariates tested
☐	☒	A description of any assumptions or corrections, such as tests of normality and adjustment for multiple comparisons
☐	☒	A full description of the statistical parameters including central tendency (e.g. means) or other basic estimates (e.g. regression coefficient) AND variation (e.g. standard deviation) or associated estimates of uncertainty (e.g. confidence intervals)
☐	☒	For null hypothesis testing, the test statistic (e.g. *F*, *t*, *r*) with confidence intervals, effect sizes, degrees of freedom and *P* value noted<br>*Give P values as exact values whenever suitable.*
☒	☐	For Bayesian analysis, information on the choice of priors and Markov chain Monte Carlo settings
☐	☒	For hierarchical and complex designs, identification of the appropriate level for tests and full reporting of outcomes
☐	☒	Estimates of effect sizes (e.g. Cohen's *d*, Pearson's *r*), indicating how they were calculated

Our web collection on statistics for biologists contains articles on many of the points above.

Software and code

Policy information about availability of computer code

Data collection	No software used.
Data analysis	R version 4.3.1. Details of data analysis,including the de novo assembled transcriptome, sequenced read counting, the downstream analysis function enrichment, the public data acquirement, orthologous analysis, metagenomic analysis, and plotting, were provided the references or repository.

For manuscripts utilizing custom algorithms or software that are central to the research but not yet described in published literature, software must be made available to editors and reviewers. We strongly encourage code deposition in a community repository (e.g. GitHub). See the Nature Portfolio guidelines for submitting code & software for further information.

Data

Policy information about availability of data

All manuscripts must include a data availability statement. This statement should provide the following information, where applicable:

- Accession codes, unique identifiers, or web links for publicly available datasets
- A description of any restrictions on data availability
- For clinical datasets or third party data, please ensure that the statement adheres to our policy

Raw RNA-seq sequencing data, de novo assembly, and RNA-Seq expression matrix in this study have been deposited in NCBI's Gene Expression Omnibus (GEO) and are accessible through GEO series accession number GSE263620. Shotgun metagenomic data generated in this study have been deposited in NCBI's Sequence Read

April 2023

Archive (SRA) with run accessions: SRR29152236 - SRR29152234. All the repositories of publicly available RNA-seq and metagenomic data used in this study are detailed in Supplementary Table 3 and Supplementary Table 4. The source data behind the graphs are available at Figshare (https://doi.org/10.6084/m9.figshare.27875763.v1). All other data are available from the corresponding author on reasonable request.

Research involving human participants, their data, or biological material

Policy information about studies with human participants or human data. See also policy information about sex, gender (identity/presentation), and sexual orientation and race, ethnicity and racism.

Reporting on sex and gender	This information was not applicable for study.
Reporting on race, ethnicity, or other socially relevant groupings	This information was not applicable for study.
Population characteristics	This information was not applicable for study.
Recruitment	This information was not applicable for study.
Ethics oversight	This information was not applicable for study.

Note that full information on the approval of the study protocol must also be provided in the manuscript.

Field-specific reporting

Please select the one below that is the best fit for your research. If you are not sure, read the appropriate sections before making your selection.

☐ Life sciences ☐ Behavioural & social sciences ☒ Ecological, evolutionary & environmental sciences

For a reference copy of the document with all sections, see nature.com/documents/nr-reporting-summary-flat.pdf

Ecological, evolutionary & environmental sciences study design

All studies must disclose on these points even when the disclosure is negative.

Study description	The shallow-water mussel Mytilus galloprovincialis were used to performed this study. All mussel were temporarily cultured on lab before experiment. 5 mussels were dissected on lab as the control group, 10 mussels were performed on 1119m cold seep environment, 5 mussels were in situ collected after 6 hours as ST group, and another 5 mussels were in situ collected after 10 days as LT group. All samples were fixed on-site.
Research sample	Mytlidae mussels are widely distributed and have adapted to various habitats ranging from shallow water to the deep-sea. Shallow-water mytilids are often used as environmental sentinels due to their extreme tolerance of various environmental stresses, Deep-sea mytilids of the subfamily Bathymodiolinae, meanwhile, are one of the dominant species found at hydrothermal vents and cold seep. In addition to their unique abilities to withstand various abiotic stresses like high pressure, low temperature and darkness, they can also build symbiotic relationships with deep-sea bacteria to obtain food and energy. Their wide distribution and ability to withstand different environmental conditions underscore their strong genetic potential for adaptation. As such, mussels serve as an ideal model species for investigating the mechanisms underlying their successful acclimatization to deep-sea environments. The shallow-waster mussel Mytilus galloprovincialis (NCBI Taxonomy ID: 29158), were widely distributed around the world. Numerous studies have revealed that shallow-water mytilids gradually adapted to cold seeps over millions of years, eventually adapting to deep-sea vents.
Sampling strategy	In May 2021, the shallow-water mussel M. galloprovincialis was harvested from the intertidal zone in Qingdao, China. These mussels were subsequently transported in coolers to the laboratory, where they underwent a month-long acclimatization process in a closed recirculating aquaculture system. This acclimatization aimed to adapt them to laboratory conditions and mitigate the stress from their collection and handling. Throughout this period, the mussels were kept in sand-filtered seawater at a salinity of 34±1‰, under a natural light cycle, and at a chilly temperature of 4°C. No food was provided during acclimatization, and the seawater was partially renewed weekly. At the end of this period, five mussels were dissected, and their gills were immediately preserved with RNAlater (Omega Biotek, USA), and flash-frozen with liquid nitrogen after being kept overnight at 4°C, thereby establishing the control group for the study.<br>The in situ experiment was carried out at Site-F cold seep of South China sea at a depth of 1,119 m, where the deep-sea mussel G. platifrons predominates as the major macrobenthic organism. Twenty shallow-water mussels were taken down to the seabed in a plastic box filled with ice, using the remotely operated vehicle (ROV) Faxian, deployed from the research vessel Kexue. To mitigate the potential of displacement by ocean currents, the container was weighted at its base. During the experiment, the container's lid was kept closed to prevent the mussels from dispersing into the surrounding environment. Perforations in the container facilitated continuous water exchange with the ambient seawater, ensuring the mussels were exposed to the deep-sea in situ conditions. Following exposure periods of 6 hours (short-term pressure: ST) and 10 days (long-term pressure: LT) to the ambient deep-sea conditions, 10 mussels were fixed using a custom-designed High-throughput in situ fixation device designed for the preservation of deep-sea macroorganisms' RNA. This device comprised four in situ fixation buckets (5L each) and a movable frame that can be mounted on the ROV. The in situ fixation buckets were pre-filled with in-house RNA stabilizing solution on board before being lowered to the underwater experimental site by ROV Faxian. The bucket was covered in the inside by a sealing rubber to avoid seawater coming into contact with the RNA stabilizing solution during the sample fixation. Prior to their placement in the fixation

device, the shallow-water mussels were gently cracked using the manipulator arm of the ROV. Following the fixation, the buckets were securely sealed by closing the lid and were then returned to the ROV's sample basket. Upon retrieval of the ROV onboard, the sampling buckets were immediately transported to the onboard laboratory. The remaining non-fixed mussels were transported onboard using insulated device. The on-site fixed mussels were immediately dissected to harvest gill tissues, which were then preserved with RNAlater (Omega Biotek, USA) and subjected to flash-freezing in liquid nitrogen following an overnight stabilization at 4°C. The preserved specimens were stored at -80°C until further analysis.

Data collection	This information was not collected.
Timing and spatial scale	This information was not collected.
Data exclusions	No data excluded from analysis.
Reproducibility	To verify the reproducibility of our findings, experiments were performed using at least five biological replicates. All attempts at replication were successful.
Randomization	Mussels were randomly allocated to experimental groups.
Blinding	Blinding was not relevant to this study because no bias could be made by the subject or the tester in the experiments performed.

Did the study involve field work? ☒ Yes ☐ No

Field work, collection and transport

Field conditions	This information was not collected.
Location	22°6.919'N and 119°17.140'; 1,119 m deep; Site-F cold seep.
Access & import/export	The in situ experiment was carried out at Site-F cold seep of south China sea at a depth of 1,119 m, where the deep-sea mussel G. platifrons predominates as the major macrobenthic organism. The shallow-water mussels were taken down to the seabed in a plastic box filled with ice, using the remotely operated vehicle (ROV) Faxian, deployed from the research vessel Kexue. Following exposure periods of 6 hours (short-term pressure: ST) and 10 days long-term pressure: LT) to the ambient deep-sea conditions, the mussels were fixed using a custom-designed High-throughput in situ fixation device designed for the preservation of deep-sea macroorganisms' RNA. This device comprised four in situ fixation buckets (5L each) and a movable frame that can be mounted on the ROV. The in situ fixation buckets were pre-filled with in-house RNA stabilizing solution on board before being lowered to the underwater experimental site by ROV faxian. The bucket was covered in the inside by a sealing rubber to avoid seawater coming into contact with the RNA stabilizing solution during the sample fixation. Prior to their placement in the fixation device, the shallow-water mussels were gently cracked using the manipulator arm of the ROV. following the fixation, the buckets were securely sealed by closing the lid and were then returned to the ROV's sample basket. Upon retrieval of the ROV onboard, the sampling buckets were immediately transported to the onboard laboratory. The in situ fixed mussels were immediately dissected to harvest gill tissues, which were then preserved with RNAlater (Omega Biotek, USA) and subjected to flash-freezing in liquid nitrogen following an overnight stabilization at 4°C.
Disturbance	Instead of collecting local species.

Reporting for specific materials, systems and methods

We require information from authors about some types of materials, experimental systems and methods used in many studies. Here, indicate whether each material, system or method listed is relevant to your study. If you are not sure if a list item applies to your research, read the appropriate section before selecting a response.

Materials & experimental systems

n/a	Involved in the study
☒	☐ Antibodies
☒	☐ Eukaryotic cell lines
☒	☐ Palaeontology and archaeology
☐	☒ Animals and other organisms
☒	☐ Clinical data
☒	☐ Dual use research of concern
☒	☐ Plants

Methods

n/a	Involved in the study
☒	☐ ChIP-seq
☒	☐ Flow cytometry
☒	☐ MRI-based neuroimaging

Animals and other research organisms

Policy information about studies involving animals; ARRIVE guidelines recommended for reporting animal research, and Sex and Gender in Research

Laboratory animals	The shallow-waster mussel Mytilus galloprovincialis (NCBl Taxonomy ID: 29158)
Wild animals	No wild animals collected.
Reporting on sex	Not available.
Field-collected samples	No wild animals collected.
Ethics oversight	No ethical approval was reguired because the study did not involve a vertebrate or cephalopod invertebrate.

Note that full information on the approval of the study protocol must also be provided in the manuscript.

Plants

Seed stocks	This study did not involve seed stocks.
Novel plant genotypes	This study did not involve novel plant.
Authentication	This study did not involve plant.
